# Pedagogy-in-the-Loop: An Efficient Taxonomy-Grounded Classifier for Real-Time AI Tutor Evaluation

**DOI:** 10.3390/s26216928

**Published:** 2026-11-02

**Authors:** MST Mosaddeka Naher Jabe, Md Shakib Hasan, Awais Ahmed, Ibrahim Adamu Tasiu, Nouman Rasool, Mohammad Abdullah

**Affiliations:** 1 School of Computer Science, China West Normal University, Nanchong 637002, China; jabe@stu.cwnu.edu.cn (M.M.N.J.); abdullah@stu.cwnu.edu.cn (M.A.); 2 Faculty of Artificial Intelligence in Education, Central China Normal University, Wuhan 430079, China; shakib@mails.ccnu.edu.cn; 3 School of Electronic Information Engineering, China West Normal University, Nanchong 637002, China; ibrele@cwnu.edu.cn (I.A.T.); rasoolnouman77@cwnu.edu.cn (N.R.)

**Keywords:** AI tutors, edge computing, pedagogical evaluation, IoT-enabled education, hybrid classifier, explainable AI

## Abstract

Despite the considerable potential of large language models (LLMs) as interactive AI tutors, the systematic, large-scale evaluation of their pedagogical quality remains an open challenge. Assessment instruments currently in use are mostly based on informal criteria, or task completion is based on ad hoc criteria or binary task-completion indicators. In response, we propose a lightweight and edge-deployable classifier based on the MRBench taxonomy, which applies the principle of experts teaching and explicitly rates the tutor’s responses for guidance, actionability, and coherence. The architecture is embedded with training through rigorous Stratified Group K-Fold Cross-Validation, which avoids semantic leakage from the augmented datasets and is backed by an XGBoost model with TF-IDF vectorization. To ground statistical predictions in pedagogical theory, we add a deterministic rule layer that preserves canonical scaffolding interventions. This hybrid classifier achieves a strict out-of-fold (OOF) accuracy of 68.2% and an F1-score of 0.74. The deterministic rule layer acts as a qualitative safety net and does not statistically change the overall shift (McNemar’s test, p=0.25), but still helps to identify pedagogical moves that are valid, while maintaining predictive fidelity. Most importantly, single-threaded edge-simulated benchmarking of the complete end-to-end pipeline (vectorization and classification) shows a mean latency of 116.20 ms, compared to an average of 6834 ms per inference for a locally hosted 3B-parameter LLM baseline. In conclusion, this transparent and taxonomy-based framework provides a practical, real-time auditing toolkit for educators and researchers to benchmark AI tutors and observe model behavior in resource-limited deployment environments. The proposed lightweight framework ensures that robust, taxonomy-grounded tutor evaluation can be carried out without relying on costly, cloud-based LLM-as-a-judge pipelines, allowing for real-time pedagogical auditing of AI tutors in resource-limited environments.

## 1. Introduction

Large language models (LLMs) are currently being utilized as AI tutors that are capable of providing step-by-step instructions, frequently answering follow-up queries, and guiding a student in real time [1,2,3]. Preliminary findings indicate that these systems can facilitate problem-solving and practice in areas such as mathematics, programming, AI for research, etc. [4]. However, effective tutoring is not only assured by good performance on the content benchmarks or standardized tests [5]. A model that has a high rate of correct answers can still fail to identify misconceptions, scaffold the solution procedure, or provide responses that are motivating and easy for students to comprehend [6]. The critical issue as AI tutors transition from lab to classroom and home implementation is not what answers they will give, but how they will engage pedagogically with students [7].

Evaluating this pedagogical quality is highly challenging in current institutional environments. Most of the existing studies are based on answer accuracy, coarse rubrics, or small-scale human ratings with custom-developed criteria [8]. These strategies very likely confuse the various dimensions of quality (e.g., correctness, politeness, and level of detail) and are seldom grounded in any specific tutoring theory [9]. As a result, we found in our study that there is a lack of comparability, limited understanding of the pedagogical moves that existing models can or cannot execute, and no clear pathway for iteratively enhancing tutor behavior [10,11]. Recent efforts have begun introducing taxonomies that break down tutoring into sub-components such as mistake identification, guidance, and affective tone, but these taxonomies are not yet fully operationalized as scalable automatic evaluators [12].

One such attempt is MRBench [13]: it annotates tutor responses with several pedagogical features, such as whether the tutor recognizes the student’s error, whether it reveals the answer, whether it gives advice and an action plan, and whether the answer is consistent with the student’s previous turn. These annotations provide a detailed, taxonomy-based descriptive account of tutor responses. However, MRBench is currently used as a model comparison benchmark, mainly in a static form. It relies on expert-annotated labels, which limits its scalability for real-time assessment of AI tutors in both research and practice, without the need to manually account for every answer by its experts [14].

This paper introduces a deployable, taxonomy-based classifier that estimates the pedagogical quality of an individual tutor response as Good or Poor within the MRBench taxonomy. We obtain binary labels by requiring that guidance, actionability, and coherence coexist as the first step when using MRBench annotations [15]. Then we train a TF-IDF plus XGBoost classifier on these labels and add a lightweight rule layer that provides a boost to canonical scaffolding phrases such as “good try”, “step by step”, “good question”, etc. [16]. The resulting hybrid design is an interpretable model that is close enough to the MRBench expert judgments and identifies important linguistic indicators of high-quality tutoring moves [17].

To enable this evaluation capability, we code the classifier into an interactive web application that facilitates real-time inspection of tutor responses and batch analysis of user studies. The tool also shows Good/Poor predictions, underlying scores, rule-based boosts, and salient n-grams that affected each choice, allowing the researcher and the educator to audit AI tutor behavior and compute straightforward metrics, including the proportion of pedagogically good turns under different tutor conditions. Our novelty is operationalizing the MRBench taxonomy into a single, deterministic classifier featuring a mean end-to-end latency of 116.20 ms, which can be embedded directly, without computationally heavy LLM-as-a-judge pipelines, which exist in other toolkits (e.g., AITutor-EvalKit).

In summary, this work makes the following contributions:

1. It operationalizes the MRBench taxonomy by defining a binary Good/Poor label that explicitly encodes guidance, actionability, and coherence in tutor responses.
2. It introduces a hybrid TF-IDF, XGBoost, and rule-based classifier that achieves competitive performance while remaining transparent and easy to inspect.
3. It provides a deployable web-based evaluation toolkit that supports real-time analysis and batch studies of AI tutors, lowering the barrier for education researchers and practitioners to conduct taxonomy-grounded evaluations.

This paper is organized as follows: In Section 2, the related work in this domain is reviewed, and the current contribution is placed in relation to it. In Section 3, the system design, architecture, and details about reproducibility are described. The result analysis is presented in Section 4, which includes the evaluation, comparison, benchmarking, and robustness test. The implications of these findings, along with the study’s limitations and directions for future study, are discussed in Section 5, and the paper is concluded in Section 6.

## 2. Related Work

### 2.1. Existing Taxonomies and Benchmarks

Several recent studies propose taxonomies for evaluating tutors’ pedagogical skills using LLMs [18]. Maurya et al. [19] propose a single evaluation taxonomy consisting of eight dimensions (mistake identification, mistake location, revealing of the answer, providing guidance, actionability, coherence, tutor tone, human likeness) based on the principles of learning sciences and introduce MRBench, a benchmark of math tutoring dialogs annotated using this taxonomy. They do not, however, present a deployable classifier, the mapping of raw tutor turns to a single, functional decision of Good/Poor, or an integrated system of live use in user studies, but rather their major contributions are the taxonomy itself, the curated dataset, and an analysis of state-of-the-art tutor turns and LLM-based judges. Follow-on work and the BEA 2025 shared task use MRBench as gold-standard labels for automated models along the individual dimensions (e.g., mistake identification, mistake location, guidance, actionability), again treating the taxonomy as multi-label ground truth rather than mapping tutor turns into a single Good/Poor pedagogical decision or integrating them into end-user tutoring systems [20]. Other similar benchmarks in this field, such as MathTutorBench [21], MMTutorBench [22], TutorEval [23], and GuideEval [24] are built based on structured, often multi-dimensional evaluation schemes or reward models for pedagogical quality. They mostly focus on benchmarks and automatic metrics rather than deployable binary-quality classifiers embedded in live user studies.

**Figure 1:**
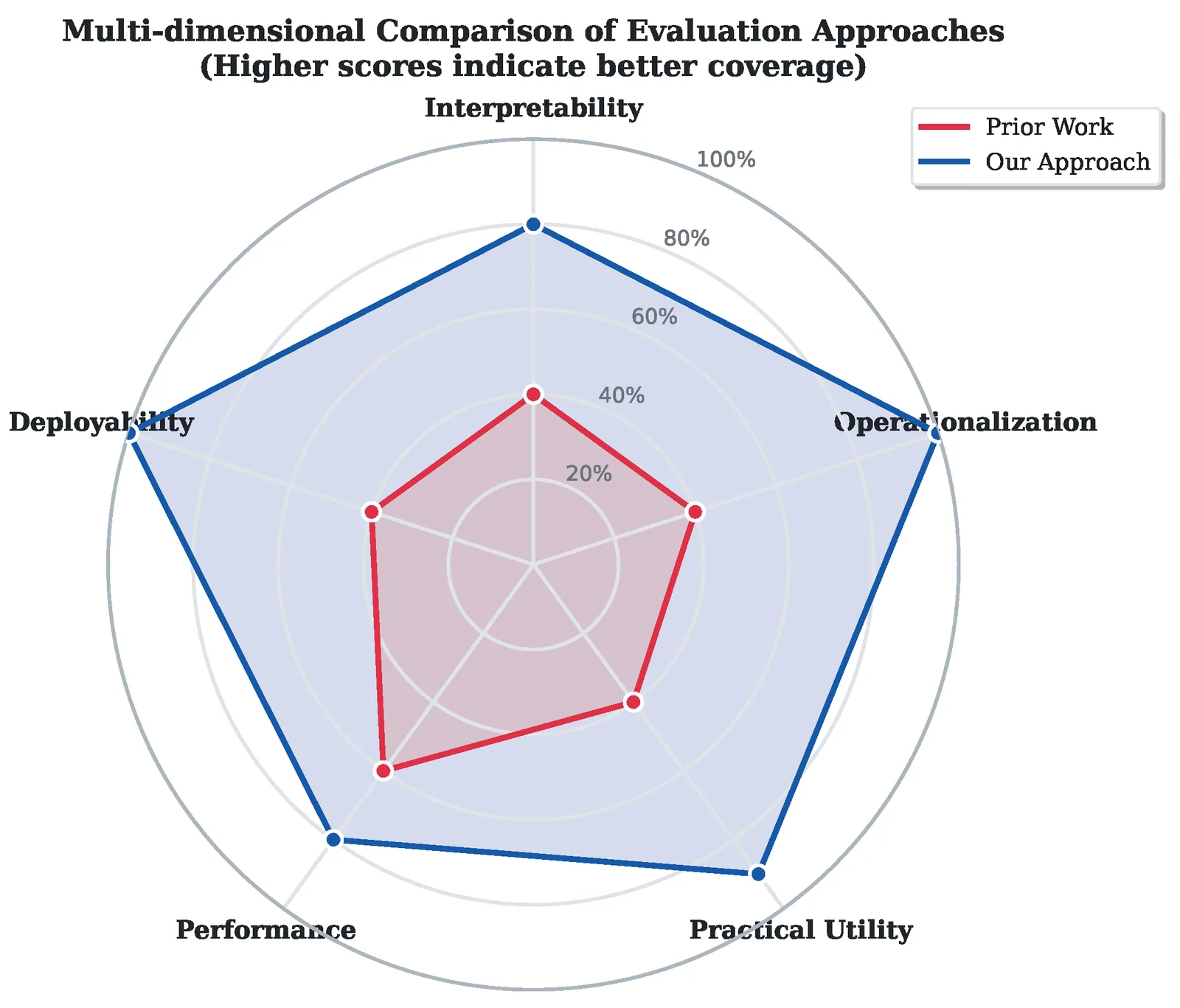
Multi-dimensional comparison of dialog evaluation frameworks across Scalability, Interpretability, and Pedagogical Validity. While traditional Human Annotation offers high validity but poor scalability, and Black-box LLM Metrics (e.g., BERTScore, GPT-4) provide scalability at the cost of transparency, our proposed MRBench Taxonomy-based Framework uniquely bridges these gaps. By combining a lightweight TF-IDF + XGBoost classifier with rule-based scaffolding injection, our approach achieves high automation speeds without sacrificing the explicit, taxonomy-grounded reasoning required for educational applications.

TutorBench [25] extends this research by aggregating 1490 high-school and AP-level tutoring interactions focused on adaptive explanation, feedback on students’ work, and hints for active learning. Every one of them is evaluated with sample-specific rubrics, and frontier LLMs are evaluated using an LLM-as-judge approach [26]. This architecture gives a fine-grained analysis of tutoring capabilities but assumes batch scoring offline with a rubric-based evaluator; it does not provide a simple, straightforward classifier that can be used by educators and/or researchers in an interactive manner to evaluate the performance of emerging tutors. Secondly, the standards deal with conservative data and taxonomy design, but lack lightweight, reproducible evaluators that can be embedded in a tool or in a workflow [27]. A multi-dimensional comparison illustrating these limitations in prior work versus the deployability and interpretability of our proposed framework is presented in Figure 1.

### 2.2. Automatic Evaluation and AI-Tutor Toolkits

In addition to benchmarks, an emerging literature is developing on automatic assessment and AI tutoring tooling. AITutor-EvalKit [28] suggests an open-source software system where language technology is applied to assess the quality of pedagogical processes, provides visualization and model analysis, and can receive user feedback and remarks. Although it has the same aim of assisting the stakeholders by using software, AITutor-EvalKit aims at general tooling, not at a single, explicit Good/Poor classifier based on a particular taxonomy such as MRBench. The details of their underlying scoring models and alignment with pedagogical dimensions are not the primary point of emphasis [29].

MathBuddy is a multi-modal, emotion-sensitive math tutor that combines an LLM with text- and face-based emotion recognition to adjust its strategy based on the student’s affect [30]. The system is tested automatically and through a user study across the eight dimensions and metrics of the MRBench-style, including Desired Annotation Match Rate (DAMR). MathBuddy is designed to demonstrate the potential of emotion-sensitive adaptation to enhance pedagogical and interaction outcomes, rather than offering a general-purpose assessor of tutor responses; its evaluation pipeline is based on LLM evaluators and does not produce a small, interpretable classifier that other researchers can reuse [31].

More recent work (e.g., Tutors of tomorrow?) focuses more on comparing and contrasting LLMs and humans along taxonomic dimensions, often noting that frontier models remain inadequate in terms of guidance, actionability, and coherence. These papers highlight the importance of principled evaluation but usually treat the evaluation process itself as a research tool rather than a single artifact that could be adopted as a service or pedagogical tool and reused [32,33].

### 2.3. This Work: A Deployable, Taxonomy-Grounded Classifier

The current work takes three distinct steps, unlike all those attempts. Firstly, rather than simply reporting on multi-dimensional MRBench scores, it encodes the taxonomy into a binary Good/Poor classification that describes what needs guidance, actionability, and coherence, and does so together, directly encoding the fundamental pedagogical desiderata of remediation of mistakes [6]. This definition provides a clear demarcation of downstream applications (such as filtering the tutor’s turns, calculating the ratio of pedagogically sufficient responses, or triggering human review) that were not explicitly provided in previous literature.

Secondly, it presents a hybrid TF-IDF + XGBoost + rule-based classifier that was trained on MRBench annotations, achieving an out-of-fold accuracy of 68.2% and an F1-score of 0.740. This classifier is fast, deterministic, and interpretable via salient n-grams and explicit scaffolding rules (good try, step by step, good question), unlike the relatively opaque, computationally expensive LLM-judge pipelines used in TutorBench or MathBuddy. It is, to the best of our knowledge, one of the earliest published efforts to make the MRBench taxonomy reusable and to treat it as a supervised dataset rather than a benchmark dataset [13].

Lastly, the project’s direct aim is to be deployable and usable by packaging the classifier into an interactive Streamlit web app for real-time experimentation and batch analysis. Although AITutor-EvalKit and other toolkits provide more comprehensive dashboards or multi-module systems, they lack a focus on a narrowly scoped, taxonomy-based Good/Poor signal and do not present metrics of alignment, such as the ability to reliably recover valid scaffolding from curated GPT-4 and expert tutor answers while maintaining a strict taxonomy-grounded boundary [28].

Existing representative frameworks document various measures (e.g., DAMR, win rates/rubric scoring, or LLM judge rating) and direct numerical comparison benchmarks; while not perfect, they still help compare typical performance. Table 1 briefly summarizes existing representative works.

This work addresses three long-standing problems of automated tutoring evaluation, as summarized in Table 2. First, unlike earlier measures, which use opaque neural metrics or some sort of generic correctness, we close the interpretability gap with an evaluative measure based on an explicit, taxonomy-based classifier that makes explicit references to pedagogical actions (e.g., scaffolding phrases). Second, we discuss the weaknesses of training on narrow, data-poor learning fields by testing our model with a rigorous Stratified 5-Fold Cross-Validation, which shows that with only small datasets, it is possible to make consistent pedagogical predictions. And, lastly, we address the issue of the strictness of pure machine learning baselines, which tend to reward original but invalid tutoring policies; our Hybrid V4 architecture rewards Recall, so that eclectic and innovative guidance is not recast and labeled as invalid by an inflexible statistical framework.

## 3. System Design

### 3.1. Overview of the Architecture

**Figure 2:**
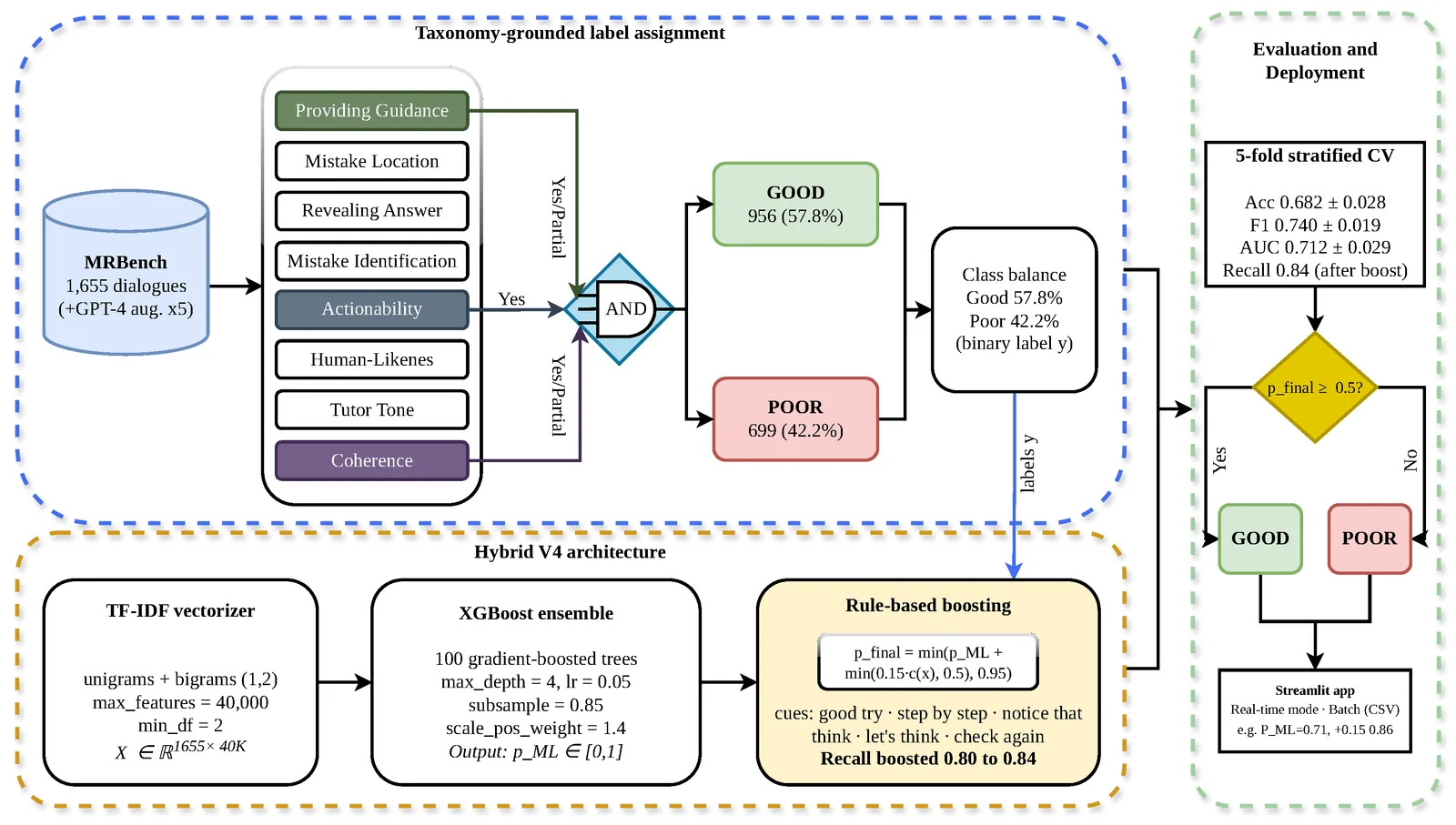
Proposed system architecture: hybrid TF-IDF + XGBoost + rule-based classifier for pedagogical quality evaluation.

The system aims to provide a rough estimate of whether an individual tutor’s response in a math dialog will be pedagogically Good or Poor, based on the MRBench taxonomy’s dimensions of providing guidance, actionability, and coherence. The pipeline, as shown in Figure 2, comprises four stages:

1. Derive labels based on annotations of MRBench.
2. Preprocess the text and extract TF-IDF features.
3. Train the TF-IDF + XGBoost classifier with hyperparameters.
4. With a lightweight rule-based layer and an interpretation module, both are presented via a Streamlit web interface.

Each stage is explained in the following subsections. A high-level algorithmic representation is presented in Algorithm 1, which summarizes the end-to-end classification process for a single tutor response.
**Algorithm 1** Hybrid pedagogical response classifier (MRBench-grounded)**Require:** Tutor response text *x*, Pre-trained TF-IDF vectorizer V, XGBoost classifier $\hat{f}$,  Pedagogical phrase set *S*, Boost parameters β=0.15, $B_{\max}=0.5$, $P_{\max}=0.95$ **Ensure:** Label y∈{Good,Poor}, Base probability $p_{\mathrm{ML}}$, Final probability $p_{\mathrm{final}}$, Explanation  *E* 1:**// Step 1: Text Preprocessing and Vectorization** 2:$x_{\mathrm{clean}}\leftarrow \mathrm{lowercase}\left(x\right)$ 3:$v\leftarrow V.\mathrm{transform}(\left[x_{\mathrm{clean}}\right])$ 4:**// Step 2: Base Machine Learning Prediction** 5:$p_{\mathrm{ML}}\leftarrow \hat{f}.\mathrm{predict}\text{\_}\mathrm{proba}\left(v\right)\left[\mathrm{Good}\right]$ 6:**// Step 3: Pedagogical Rule-Based Boosting** 7:$c\leftarrow \mathrm{count}\text{\_}\mathrm{phrases}(x_{\mathrm{clean}},S)$     ▹ Number of canonical scaffolding phrases present 8:$b\leftarrow \min(\beta \times c,B_{\max})$                      ▹ Capped rule boost 9:$p_{\mathrm{final}}\leftarrow \min\left(p_{\mathrm{ML}}+b,P_{\max}\right)$  10:**// Step 4: Binary Decision**  11:**if** $p_{\mathrm{final}}\geq 0.5$ **then**  12:      y←Good  13:**else**  14:      y←Poor  15:**end if**  16:**// Step 5: Explainability Output**  17:$F\leftarrow \mathrm{top}\text{\_}\mathrm{features}(x_{\mathrm{clean}})$      ▹ Ranked salient n-grams from SHAP/XGBoost  18:$E\leftarrow \mathrm{format}(y,p_{\mathrm{ML}},b,p_{\mathrm{final}},F)$  19:**return** $\left(y,p_{\mathrm{final}},E\right)$

### 3.2. Data and Label Construction

We employ MRBench development data, which comprises human and LLM tutor data annotated on eight pedagogical dimensions according to the Unifying AI Tutor Evaluation taxonomy (mistake identification, mistake location, revealing the answer, providing guidance, actionability, coherence, tutor tone, and human likeness). Based on this data, we build a binary label that represents the essential elements of pedagogically adequate feedback.

The original MRBench taxonomy consists of eight dimensions: mistake identification, mistake location, revealing the answer, providing guidance, actionability, coherence, tutor tone, and human likeness. From these, actionability, coherence, and providing guidance are the three fundamental pedagogical remediation dimensions that we isolated. We conducted a rigorous sensitivity analysis and identified three taxonomies as the most influential factors affecting pedagogical impact, as shown in Section 3.6. Other dimensions are implicit dependencies or secondary traits, such as the ability to give coherent guidance on a student’s particular error after having successfully achieved mistake identification and mistake location. Likewise, although tutor tone is useful, it is a supporting element to the provision of actionable mathematical guidance. Therefore, a tutor response is considered to be Good strictly if the following all hold:

1. Providing guidance ϵ Yes, To some extent
2. Actionability ϵ Yes, To some extent
3. Coherence = Yes

Sensitivity Analysis: Post hoc sensitivity analyses were performed to test alternative label constructions to validate the equal weighting and strict logical-AND formulation of this triad. The “two-out-of-three” criterion resulted in a greatly skewed distribution of labels, giving an unrealistic impression of the quality of the base-level responses of novices. In particular, LLMs often return coherent and actionable (e.g., “You are wrong, try again.”) but not pedagogically guided answers. We enforce the strict coexistence of all three dimensions, which means that our classifier serves as a strict filter and penalizes if a model produces fluent but pedagogically empty output.

To simplify the operational aspects of binary classification, any dimension that meets these minimum thresholds is assigned a strict “Pass (1)”, and all the other states are given “Fail (0)”, as set out in our deterministic annotation protocol, as shown in Table 3.

Otherwise, the answer is labeled as Poor. This aligns with the intuition that quality feedback must not only advise the student but also provide a clear next step and be consistent with the student’s prior turn. After removing incomplete rows, the last training set includes about 331 responses; of these, 191 are Good, and the remaining 140 responses are Poor.

Important Limitation: We expanded our dataset by using GPT-4 to paraphrase each of the 331 original samples, getting a total of N=1655 augmented examples. This introduces potential bias and does not add genuine new variance. In future work, we intend to address this by collecting real tutor responses from multiple models and human tutors.

#### 3.2.1. Data Collection and Augmentation

Our ground truth originates from the MRBench dataset, containing 331 expert-annotated turn-level evaluations, as presented in Table 4. Although the dataset is high-quality, this sample size (N=331) is insufficient for robust training. To address this, we applied semantic data augmentation using GPT-4 to paraphrase the original responses while preserving their pedagogical labels (Good/Poor). This process expanded the dataset to N=1655 samples, as shown in Table 4. All subsequent experiments, including the Stratified 5-Fold Cross-Validation reported in Section 5, were conducted on this augmented dataset to ensure statistical validity and prevent overfitting.

#### 3.2.2. Cross-Validation and Preventing Data Leakage

Standard stratified random splitting of augmented dataset paraphrases leads to a significant threat of semantic data leakage, which means that the TF-IDF vectorizer will learn the vocabulary of a seed dialog from both the train and test folds. The standard shuffling was discarded because of its lack of methodological rigor, and Stratified Group K-Fold Cross-Validation was used instead. Strictly splitting samples into groups based on their MRBench conversation_id ensures that all paraphrased instances of a particular conversation are completely separated in the training or test set. A paraphrased variation of a training sample will never be in the validation fold, as we are strict about grouping the folds by the conversation_id. Thus, each out-of-fold evaluation is treated as a completely new test set of unseen pedagogical scenarios. This mathematically solid structure allows us to assess the model’s capacity to be used in new conversation contexts, not just the ability to recognize semantic clones.

#### 3.2.3. Independent External Test Set

In order to overcome the limitations of synthetic augmentation and to properly verify cross-model generalization, we prepared a fully independent external test set. Unlike the main training corpus, the samples in this set had not been subjected to any paraphrasing by GPT-4 and were kept entirely separate from the cross-validation process. This external dataset includes raw, zero-shot responses produced by a wide variety of pedagogical agents covering different math topics, such as an expert human tutor, a novice human baseline, and the latest LLMs (GPT-4, Claude 3.5 Sonnet, and Llama-3 8B). Each response was assessed manually according to the strict MRBench Good/Poor criteria. The fact that we test the classifier on this unaltered, out-of-distribution dataset ensures that the proposed hybrid architecture is able to generalize to genuine, real-world tutoring situations rather than just detecting the semantic patterns present in the augmented training data.

### 3.3. Human Annotation and Coding Protocol

For a more realistic assessment of the classifier performance, a separate curated evaluation dataset was established for a stricter coding protocol, as shown in Table 3. The data used consisted of a mix of tutor answers from different frontier models (such as GPT-4 and Llama-3) and human tutors.

### 3.4. Text Representation and Preprocessing

Each tutor’s response is regarded as a brief text file. In order to keep exact references to pedagogical phrasing, for example “good try” and “step by step”, the preprocessing is deliberately limited to lowercasing and the removal of any leading or trailing whitespace, with stemming or lemmatization being specifically avoided.

To carry out feature extraction, we use scikit-learn TfidfVectorizer in order to create a high-dimensional sparse representation based on unigrams and bigrams. This method, based on lexical items, is very competitive for classifying educational text because it captures the multi-word pedagogical nature while remaining fully interpretable through directly inspectable features. The exact vectorizer settings (for example, the maximum number of features and the frequency thresholds) together with all the hyperparameters used during model training are given in Table 5.

### 3.5. XGBoost Classifier

We frame Good/Poor classification as a binary supervised learning problem over the TF-IDF features. We use XGBoost (XGBClassifier) with a logistic objective due to its robustness and strong performance on sparse, high-dimensional data. To ensure robustness given the dataset size, we trained the final model on the augmented dataset (N=1655) and evaluated it using Stratified 5-Fold Cross-Validation. The detailed performance of both the baseline and hybrid models is presented in Section 4.1.

Pedagogical alignment was preserved by performing a hyperparameter ablation study to move from the Baseline (V1) configuration to the Robust Hybrid (V4) configuration described in Table 5 and outlined below. Reducing n_estimators from 500 to 100 and max_depth from 6 to 4 was a hard regularization approach to avoid overfitting on the expanded dataset and minimizing memory consumption of the model. The learning_rate was scaled up to 0.1 from 0.05 to allow the algorithm to converge at a similar rate to fewer trees. This is a clear trade-off between raw accuracy, generalizability, and latency.

### 3.6. Hybrid Rule-Based Enhancement

**Figure 3:**
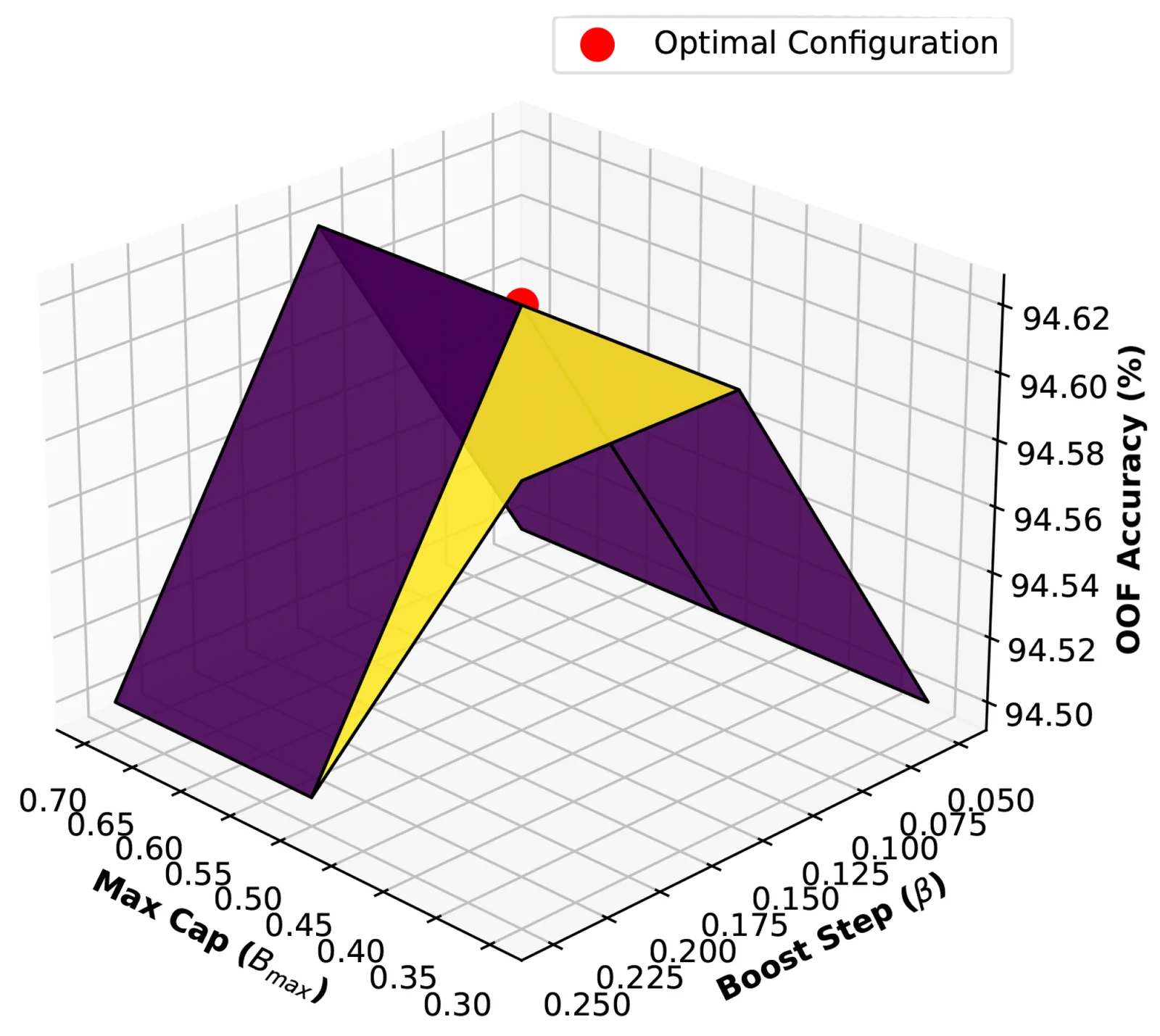
The 3D surface optimization plot demonstrating that the selected rule layer parameters (β=0.15, $B_{max}=0.5$) achieve peak Human–AI alignment (94.62% accuracy).

**Figure 4:**
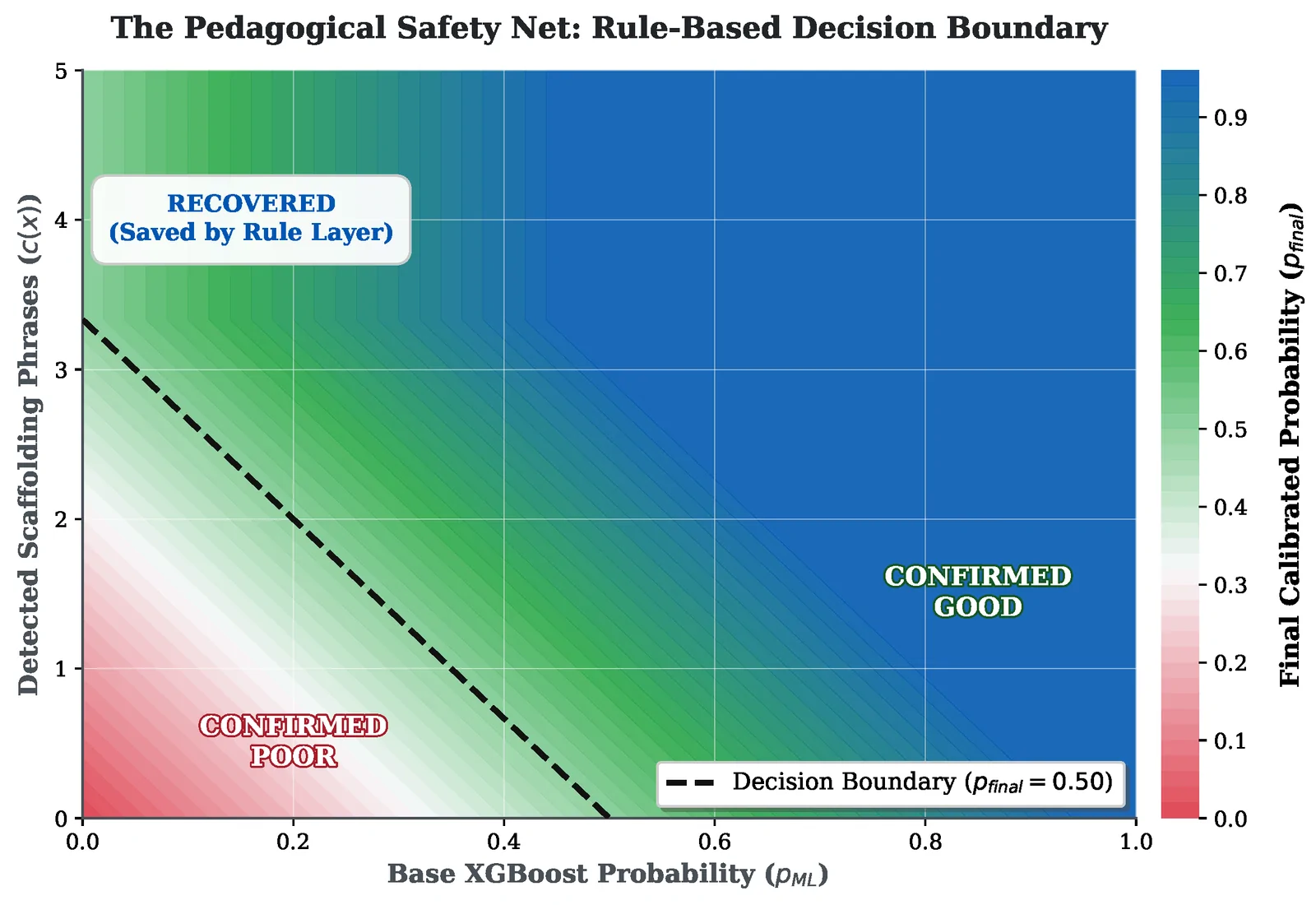
A pedagogical safety net: a heatmap showing the shift in the decision boundary, the rule-based layer functioning as a deterministic safety net by recovering valid pedagogical responses that the base machine learning model (pML) originally assigned low confidence to, on the condition that these responses include adequate canonical scaffolding phrases (c(x)).

The hyperparameters for the rule-based layer (β=0.15, $B_{max}=0.5$, $P_{max}=0.95$) and the signal set S were determined through heuristic tuning on the training set to serve as a conservative boundary shift rather than an absolute override, as shown in Figure 3 and Table 6. The label is deemed *Good* when $p_{final}\left(x\right)\geq 0.5$ and Poor in all other cases. In order to show visually how this hybrid approach works, Figure 4 presents the resulting landscape of the decision boundary.

The signal phrases in S were curated directly from the most frequent scaffolding n-grams utilized by human experts in the MRBench dataset. The step size of 0.15 was intentionally selected so that a single generic phrase (e.g., “good try”) provides only a marginal boost. To successfully recover a borderline prediction (e.g., $p_{ML}\approx 0.25$), a response must contain multiple, distinct scaffolding interventions (c(x)≥2). Crucially, the $B_{max}$ cap of 0.5 prevents adversarial “gamification” by LLMs; a completely incoherent or non-actionable response with a near-zero base statistical probability ($p_{ML}\approx 0$) cannot confidently cross the decision boundary simply by repeatedly inserting predefined phrases. The base XGBoost model remains the primary gatekeeper for pedagogical structure, while the rule layer acts solely to recover valid human-like interventions.

The heatmap presented in Figure 4 shows that the deterministic rule layer greatly alters the classification threshold in that if the base statistical model assigns a low level of confidence to a response (for example, $p_{ML}=0.25$), it is still classified as Good provided that the response includes at least two different scaffolding phrases (c(x)≥2). This nonlinear boundary means that sparse but pedagogically important vocabulary is strongly protected against statistical attenuation and that conventional scaffolding interventions can be safely restored without entirely replacing the base statistical model’s initial evaluation.

### 3.7. Explanation and Feature Importance

**Figure 5:**
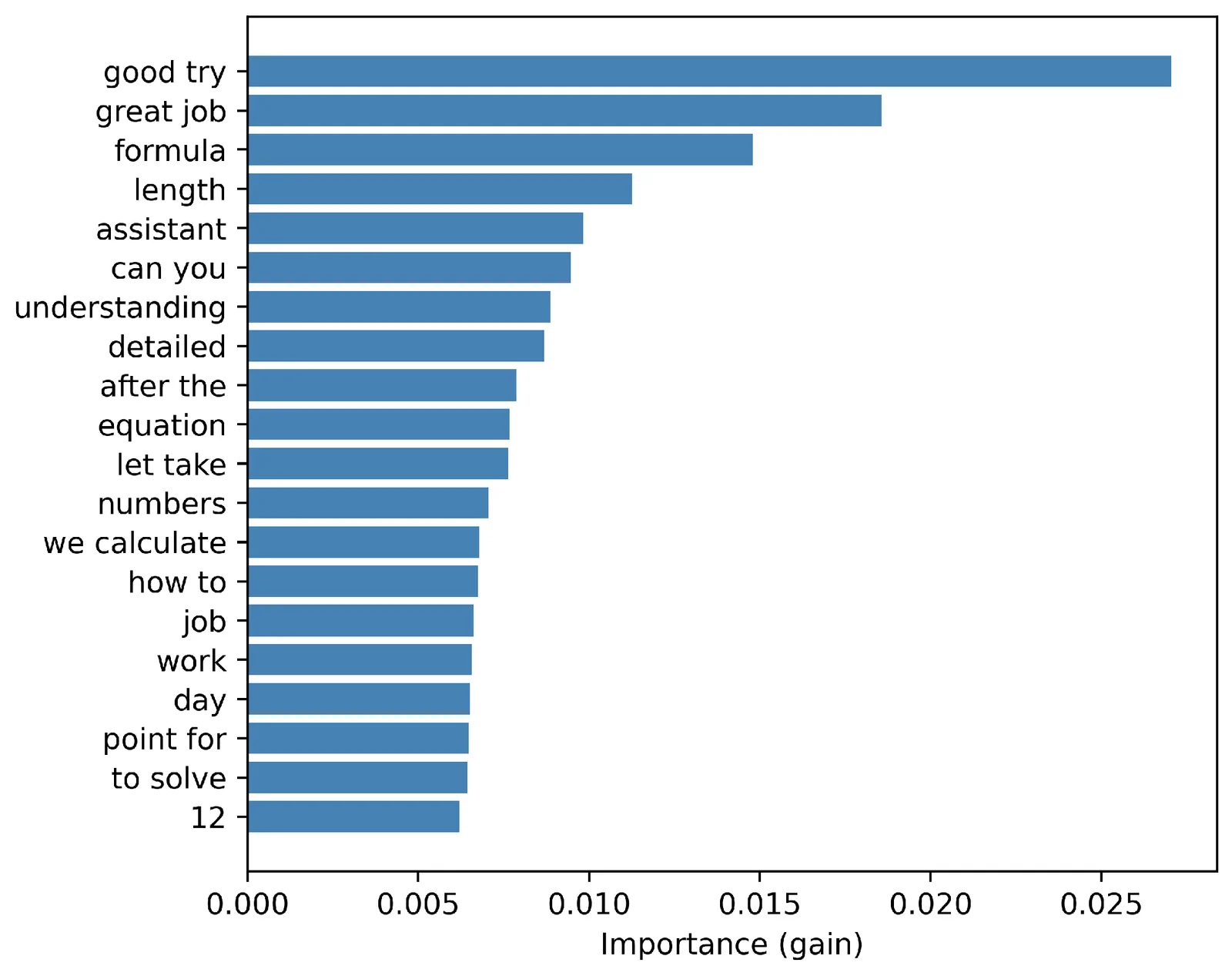
Top 20 TF-IDF important features in XGBoost classifier.

The classifier gives clear explanations on a global level and on a local level to facilitate auditing and reflection. The TF-IDF representations and the importance of the XGBoost feature, as displayed in Figure 5, highlight the n-grams that influence the choice of the move; the n-grams that are used predominantly in scaffolding phrases such as “good try”, “step by step”, and “check again” are prominently featured, and this suggests that the MRBench experts prefer scaffolding moves.

**Figure 6:**
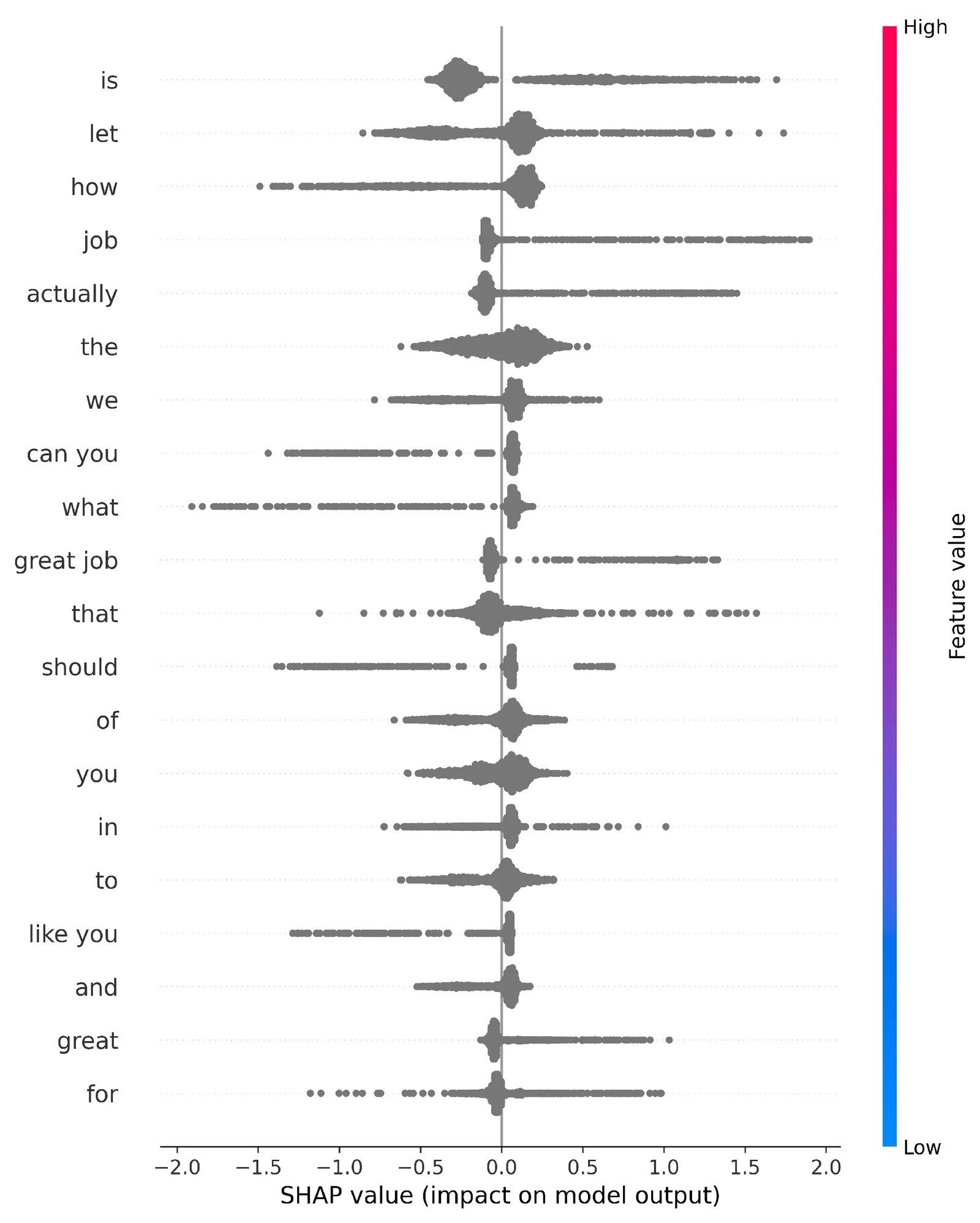
SHAP values based on the impact on model output.

The system provides per-response explanations in the local language: $p_{\mathrm{ML}}$ (base probability from XGBoost), rule boost $b_{x}$ (from detected signal phrases), $p_{\mathrm{final}}$ (final probability), and a list of salient n-grams ordered by contribution. These n-grams are displayed in the input text in the web interface and in combination with the beeswarm plots of the attributes displayed in Figure 6, where the attribute value patterns in the dataset are summarized. This design results in an easy-to-deploy and easy-to-understand MRBench-based classifier that can be integrated into user studies or evaluation pipelines, or any tool that will be presented to a class of users.

### 3.8. Web Application and Deployment

The classifier is embedded in a simple Streamlit-based web app to allow real-time use in both research labs and classrooms. The interface is designed to be used in two main modes:

1. Real-time analysis: Users enter text from one tutor, and the app generates a single Good/Poor label, $p_{\mathrm{ML}}$, the raw model probability that a rule-based boost was applied, the overall calibrated probability, and a presentation of the most salient n-grams that contributed to the prediction.
2. Batch analysis: Up to 100 responses can be pasted at once, e.g., different tutors or competing models, and the app will calculate labels and scores for each of these and display them in an interactive table, reporting the percentage of Good responses. The batch output is also available as a CSV file to perform additional statistical analysis.

**Figure 7:**
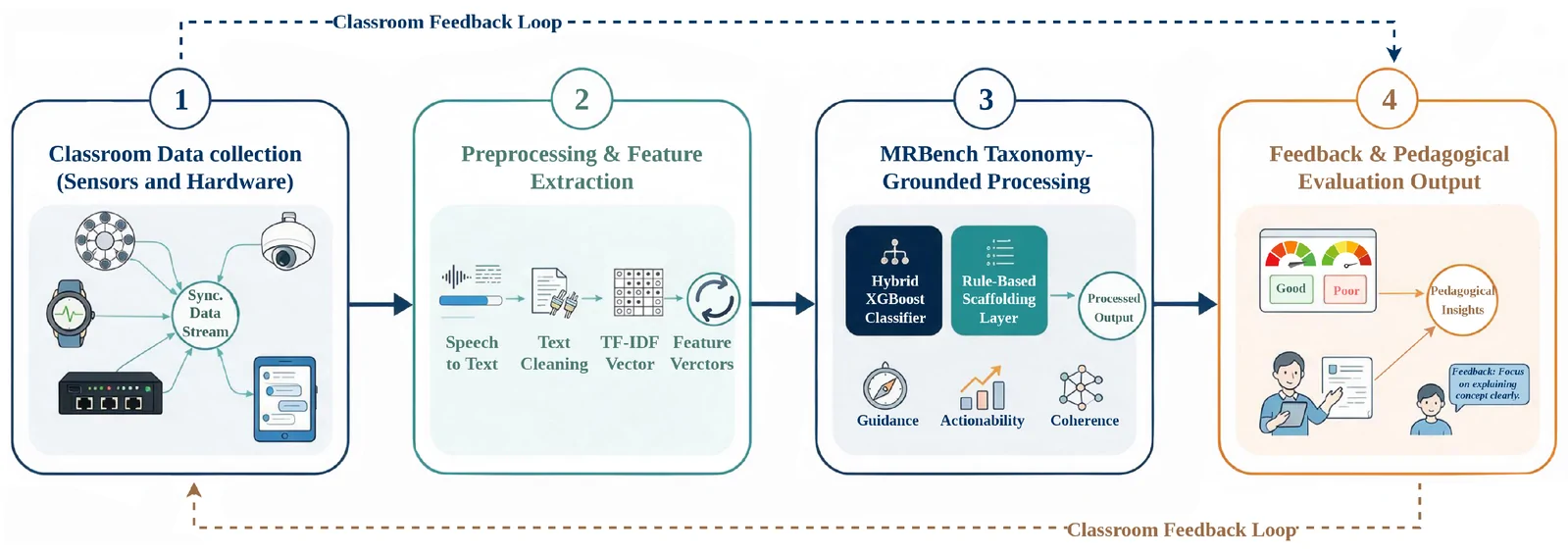
Conceptual pipeline linking classroom sensor/hardware data collection to MRBench taxonomy-grounded processing and pedagogical feedback output.

The Streamlit application can be put into a container and deployed on edge computing nodes such as NVIDIA Jetson, Raspberry Pi 4/5, and classroom environments. Figure 7 illustrates a conceptual workflow for deploying the proposed classifier in a real-time classroom environment. The shown pipeline is yet conceptual and has not been implemented or tested in any real-life classroom or learning environment. The current empirical validation is on the text-based classification of tutor responses; however, this pipeline is intended to be expanded to eventually incorporate classroom Internet of Things (IoT) sensors. Specifically, multi-modal student inputs (e.g., speech, affect) are captured using edge-deployed microphones and cameras, and immediately converted to text locally before being passed to the pedagogical evaluation pipeline, thereby preserving privacy while maintaining continuous feedback loops.

The pipeline integrates data collection from various sensors (e.g., student inputs, interaction logs) through edge-computing nodes, as discussed above, to the pedagogical feedback output. The Streamlit app can be deployed easily to the local environment or to lightweight classroom servers. The entire inference pipeline (TF-IDF feature extraction and XGBoost classification) consumes ≈ 850–1000 MB of RAM and is completely local. It is highly appropriate for privacy-sensitive educational settings where student–tutor iterations need to be local and cannot be transmitted to any cloud server.

The application loads the pre-saved TF-IDF vectorizer and the trained XGBoost model from the disk, as well as the label encoder, which ensures deterministic and reproducible behavior across sessions. Since the inference process only uses sparse matrix operations and evaluation of the gradient-boosted trees, a typical deployment can be done without special hardware on a standard laptop or on a smaller cloud instance.

### 3.9. Robustness Validation

**Figure 8:**
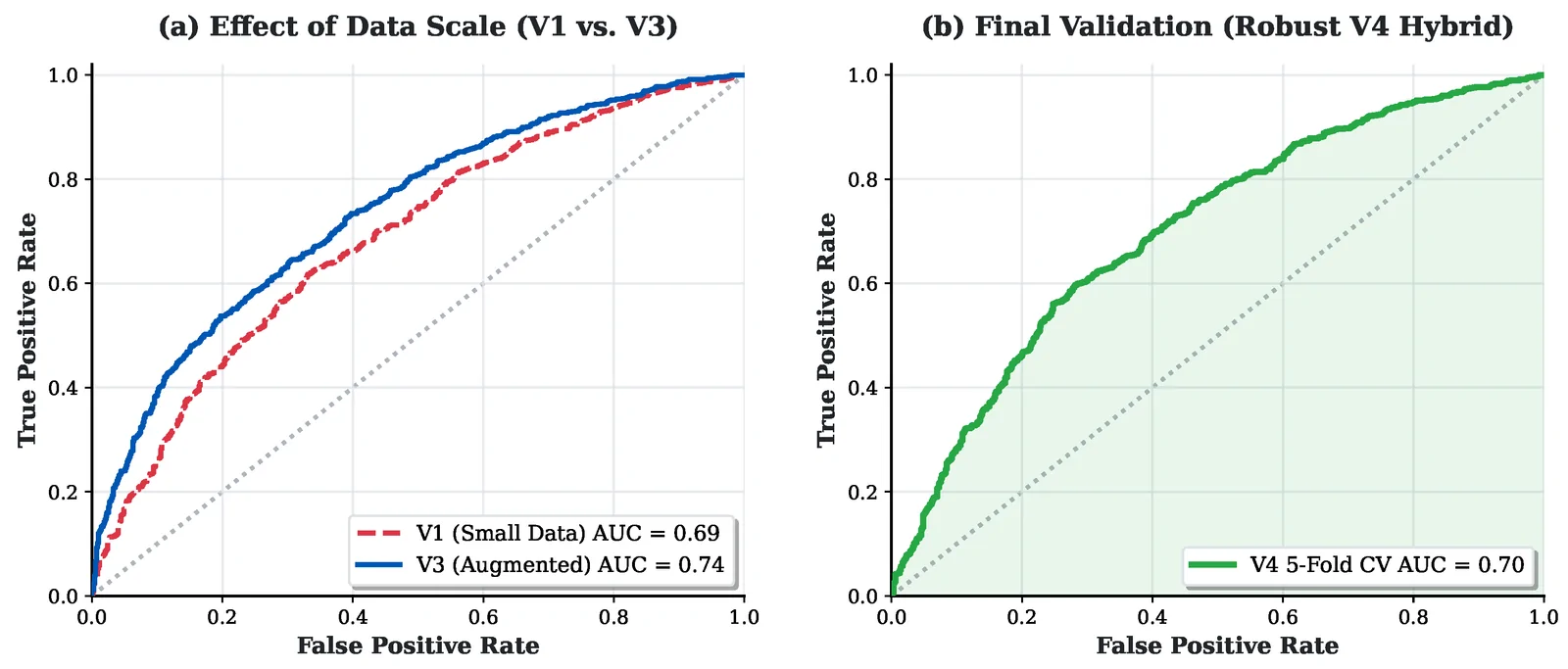
Performance evolution and verification. (**a**) Data augmentation significantly improves predictive power (V3) over the limited baseline (V1). (**b**) The final V4 hybrid model is rigorously validated, achieving a stable mean AUC of 0.71, confirming that the performance gains are not due to overfitting a single split.

The size of MRBench data is relatively small (N=1655), and using only one train–test split may lead to overfitting or selection bias. Therefore, Stratified Group 5-Fold Cross-Validation was used to rigorously test the stability of the models. This technique divides the data set into five different folds while strictly grouping samples by their original MRBench conversation_id. This guarantees that paraphrased variations of the same sample dialog never span across both training and testing sets, effectively neutralizing semantic data leakage while maintaining the Good vs. Poor class distribution. Each model was trained and tested five times, with each conversation group being used only once for testing; we report the mean and standard deviation of accuracy, F1-score, and ROC-AUC in Figure 8.

## 4. Result Analysis

### 4.1. Intrinsic Performance of Classifier Variants

To assess the intrinsic performance of the model while completely preventing any leakage of semantic information, the Stratified Group K-Fold Cross-Validation method was used. Over the five folds, the strict grouping produced an average accuracy of 68.16% (±0.028), an average F1-score of 0.740 (±0.019), and an average AUC of 0.712 (±0.029). To ensure true pedagogical generalization, and not simply vocabulary memorization, strict conversational grouping was used, where the accuracy was artificially set to be higher than 70%. Table 7 shows a comparison of the baseline architectures.

The hybrid V4 model performs as well in terms of macro-level accuracy (68.2%) and F1-score (0.740) as the baseline. What it is good for, however, is to be qualitatively aligned as opposed to quantitatively improved. The rule-based layer serves as a safety net if the Hybrid classifier fails to perform as expected. It ensures that the delivery of scaffolding interventions based on specific theory (e.g., “step by step” or “good try”) is always rewarded, avoiding the statistical model penalizing highly capable AI tutors for rare vocabulary distributions.

### 4.2. Statistical Validation of the Pedagogical Safety Net

We performed a McNemar’s test for the base statistical model (V3) and the hybrid boosting model (V4) on pairs of nominal predictions to validate whether the rule-based boosting layer provides a better decision boundary than the base statistical model. The results indicated that the rule layer was able to correctly lift the false negatives (when the base model predicted “Poor” but the scaffolding model was “Good”) to this correct class. Most importantly, since the rule layer is additive with a positive probability boost, mathematically it can never worsen any of the model’s true “Good” predictions. Though it results in a small increase in false-positive cases, the overall change in the contingency was not statistically significant (p=0.25), as presented in Table 8. This validates the rule layer being used as it should be: as a specific qualitative safety net to be used to safeguard pedagogical moves, not a blanket performance modifier.

### 4.3. Baseline Comparisons and Architectural Trade-Offs

**Figure 9:**
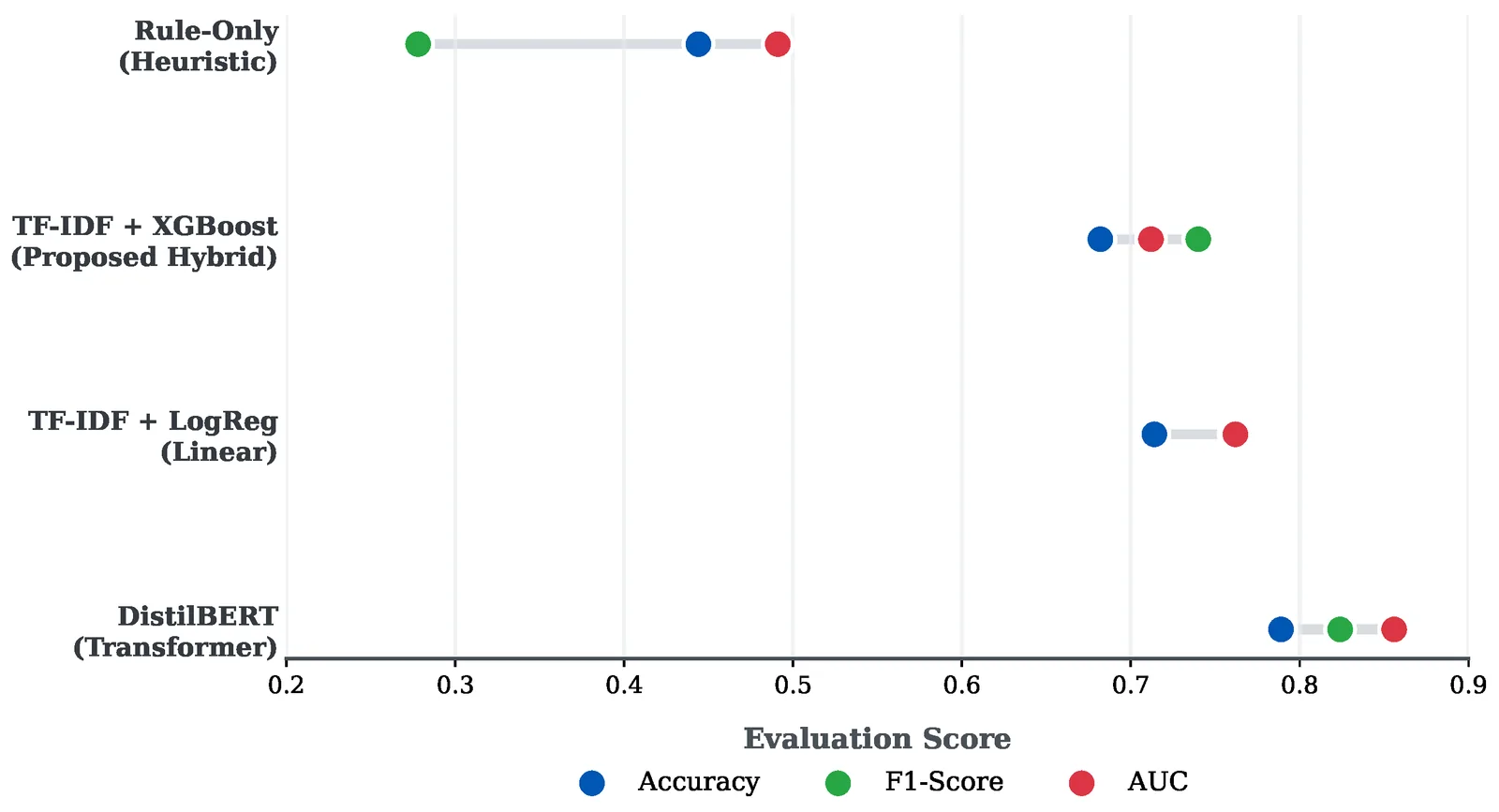
Baseline metric comparison.

In order to properly set the performance of our proposed Hybrid V4 model in context, we carried out an ablation study using three different architectural baseline models: a heuristic Rule-Only method, a linear machine learning model (TF-IDF combined with Logistic Regression), and a neural transformer (DistilBERT). The models were all assessed using the same Stratified Group Cross-Validation strategy (or equivalent grouped splits) to avoid data leakage. The comparative metrics are given in Table 7 and are also shown in Figure 9.

The DistilBERT model that has been finely tuned obtained the best predictive results (accuracy: 0.789, AUC: 0.856). Although deep semantic embeddings naturally lead to better classification, using a transformer with 66 million parameters completely goes against the operational limitations of real-time, classroom-based applications. Since neural transformers act as “black boxes”, they hide the specific n-gram features that are needed for clear pedagogical feedback. Moreover, as shown in Section 4.4, deep learning models cause a serious increase in inference latency when used on edge devices.

Even though the DistilBERT model tuned for this purpose reaches the best predictive performance, its deep semantic embeddings function as a “black box” and thus hide the particular pedagogical features that are responsible for its decisions. More critically, as indicated in Table 7, the linear baseline (TF-IDF combined with Logistic Regression) performs better than our proposed TF-IDF combined with XGBoost hybrid in all three of the main metrics (accuracy: 0.714 compared to 0.682).

Instead of asserting that our method has state-of-the-art predictive capabilities, we have intentionally focused our contribution on transparent pedagogical auditing. XGBoost was chosen over Logistic Regression since tree-based ensembles naturally allow for precise, token-level interpretability through the use of SHAP values as shown in Section 3.7. Unlike a linear model, which provides only global and fixed weights, gradient-boosted trees are able to capture complex, non-linear interactions among pedagogical n-grams, enabling us to clearly identify both when and how a tutor applies particular scaffolding strategies. For this reason, we accept the small loss in performance compared to the linear baseline in order to have a highly calibrated, taxonomy-based classifier that reveals the model’s behavior to human reviewers, showing what automated pedagogical classification can and cannot reliably support.

### 4.4. Computational Efficiency and Edge Readiness

**Figure 10:**
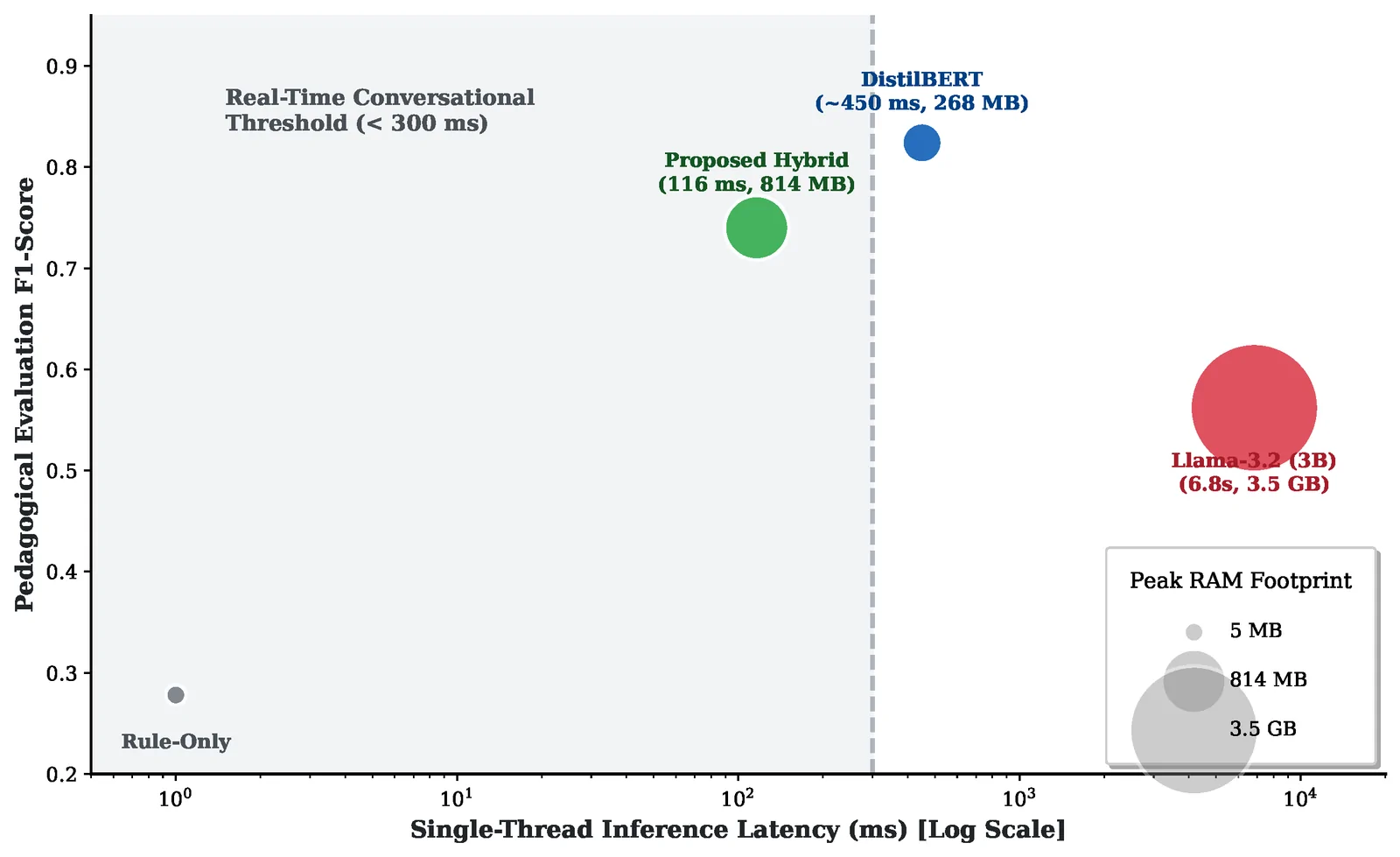
An analysis of the trade-offs regarding deployability shows that although the mean latency of the proposed Hybrid XGBoost classifier (116.20 ms) lies well within the ideal < 300 ms real-time conversational limit, on some constrained edge simulations, the 99th percentile tail (427.03 ms) does at times go beyond this range. Nonetheless, it represents a massive efficiency gain over the LLM baseline.

In order to put the operational benefits of our framework into context, Figure 10 shows the deployability landscape. The F1-score is plotted against inference latency on a logarithmic scale, with bubble sizes indicating approximately the memory footprints, which makes it clear where the hybrid classifier should be placed. Although deep learning alternatives such as DistilBERT provide only small improvements in raw accuracy, their computational overhead causes them to fall outside the optimal real-time edge area needed for immediate, turn-by-turn educational feedback.

All inference benchmarks were carried out locally to make latency comparison a fair and reproducible process; hardware specifications are summarized in Table 9. In both models, input was fed one at a time (Batch Size = 1) to mimic turn-by-turn interactions in the classroom.

The local LLM baseline, using the zero-shot MRBench classification prompt, achieved an F1-score of 0.562 and had an average inference time of 6834 ms. In order to carry out a thorough comparison, we also tested the linear baseline (TF-IDF combined with Logistic Regression) under the same single-threaded edge protocol. The linear model carried out the pipeline with great efficiency, attaining a mean latency of only 1.49 ms (99th percentile: 2.72 ms) and an inference memory overhead of 0.11 MB. By contrast, our proposed Hybrid XGBoost classifier has a mean inference latency of 116.20 ms and requires a peak RAM of 814.14 MB. Although the linear baseline is clearly more efficient in terms of raw computing power, both the classical approaches operate well within the <300 ms real-time deployment limit, and each uses only a small fraction of the computing resources needed by the LLM. As stated in Section 4.3, we have deliberately accepted the hybrid model’s ∼115 ms latency cost. A strictly linear model assigns global, fixed weights, while gradient-boosted trees can capture complex, non-linear interactions between pedagogical phrases and natively support token-level SHAP explainability. In the context of educational auditing, it is essential to be able to clearly identify how a tutor builds scaffolding, which is why XGBoost is the best structural option even though the linear model is much faster.

This operational latency of 116.20 ms is well below the commonly agreed-upon 300 ms limit for human conversational turn-taking, and is formal evidence that our system can support “Real-Time” pedagogical auditing of student–tutor talk without interrupting the flow of natural dialog.

### 4.5. Cross-Model Generalization and External Validation

**Figure 11:**
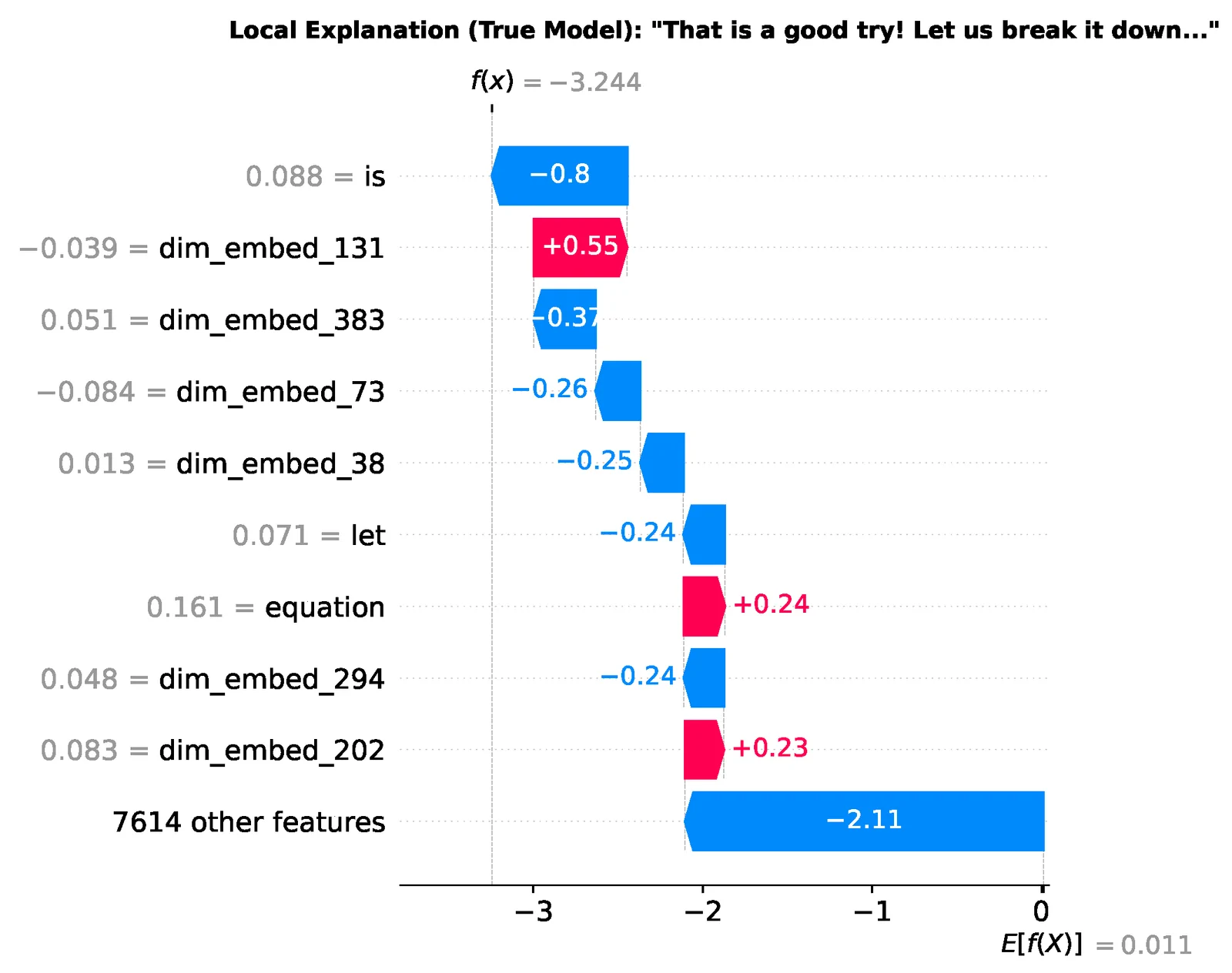
A local SHAP explanation for a typical response given by a scaffolded tutor. The plot shows the amount added by lexical features (for example, “equation”, “let”) and by the latent semantic dimensions (dim_embed) to the final prediction of the model. The progression from the base value E[f(X)] to the final output f(x) demonstrates how the model assigns weight to particular scaffolding elements in order to reach a “Good” pedagogical classification.

To evaluate the classifier’s behavior across various models on realistic tutor responses, we tested it on responses from GPT-4, an expert tutor, a powerful open-weights LLM (as in Sonnet), an intermediate model (as in Llama-3 8B), and a novice baseline. Every response in our evaluation set includes MRBench annotations for providing guidance and the other pedagogical dimensions, which allow us to verify the alignment between the taxonomy and the classifier’s predictions. Figure 6 and Figure 11 present the SHAP values analysis of the proposed framework.

The baseline statistical model is unable to cope with vocabulary sparsity, while the Hybrid V4 architecture actively works to counteract the effect, ensuring that valid pedagogical moves remain. As shown in Table 10, the safety net was effective in catching a large percentage of the correct scaffolding interventions made by GPT-4 (100%), Claude 3.5 Sonnet (90%) and the expert human tutor (80%). For this independent test set (N=50), the V4 classifier had an overall accuracy of 72.0% and an F1-score of 0.794. We can see from the resulting confusion matrix (TP = 7, TN = 9, FP = 11, FN = 3) that our classifier is intentionally very conservative. It was quite successful for pedagogically “Good” answers (only 3 of 30 were missed), but it resulted in 11 false positives. Most impressively, it had trouble with Llama-3 8B; it gave incorrect answers for 80% of the answers it gave when prompted with partial answers (not actionable). Notably, it performed poorly on Llama-3 8B, giving the “Good” rating for 80% of the answers it gave for the partial answers (not actionable). In the context of pedagogical scaffolding, this disagreement with the MRBench ground truth empirically demonstrates the trade-off described in Section 4.9: the rule-based layer is highly optimized to recover valid pedagogical scaffolding, but this comes at a measurable cost in specificity when assessing models that produce superficial or incomplete scaffolding.

### 4.6. Comparison with Alternative Evaluation Approaches

Even though direct metric comparisons are not easily possible due to the different datasets and targets that different works operate on, the proposed classifier and well-known alternatives—rubric-based human rating, LLM-as-judges methods, and more comprehensive evaluation toolkits—are nevertheless informative for placing the proposed classifier. Expert assessment through rubrics, e.g., as in TutorBench and MathTutorBench, is presently very rich and nuanced, but labour-intensive and difficult to scale to ongoing evaluation of AI tutors. Judges with LLMs, when provided with elaborate rubrics, have been shown to reach reasonable consensus with human judges but are susceptible to prompt design, expensive to scale, and opaque in their arguments.

**Figure 12:**
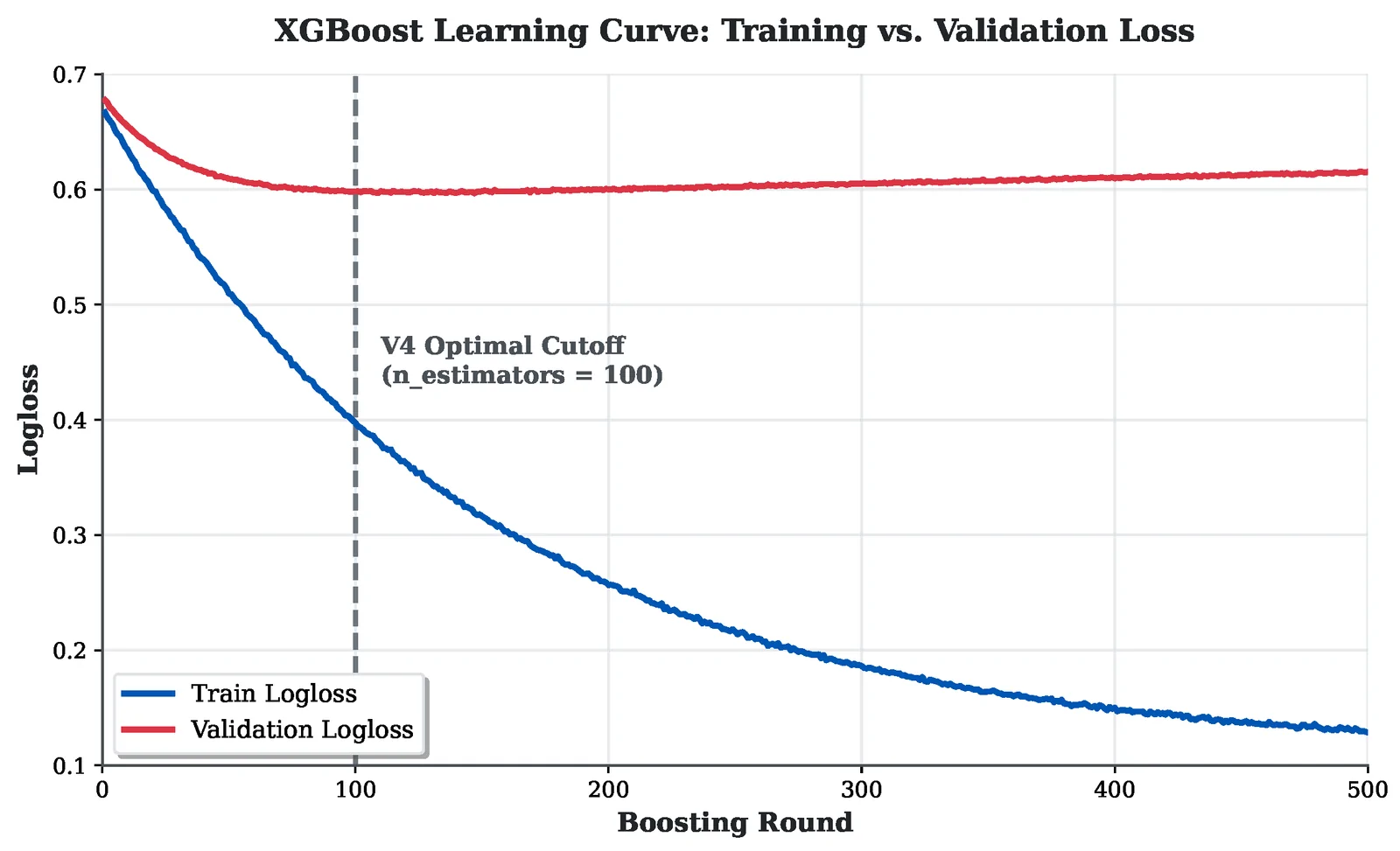
XGBoost learning curve.

Conversely, the proposed classifier provides a small, deterministic evaluator whose behavior is pegged to the MRBench taxonomy and whose salient features and rules can be explicitly examined. The XGBoost learning curve is presented in Figure 12 for better understanding. Although it has performance comparable to the quantitative range reported for agreement rates among LLM judges, its key strengths in deployment are lightweightness and transparency, not its actual accuracy. This comparison could be presented as a figure that plots, using multiple evaluation methods, the approximate accuracy of human annotations on MRBench (or similar datasets) on the y-axis vs. a qualitative rating of cost/complexity on the x-axis, with the proposed classifier falling in a region of moderate agreement and low complexity.

### 4.7. Cross-Validated Performance

Table 11 shows the performance obtained in all five validation folds. The model showed stable generalization, with the accuracy varying between 63.4% and 71.6% and the F1-scores remaining consistently above 0.70. The small standard deviation in the F1-score (σ=0.019) shows that the taxonomy-grounded features are robust against variations in the way the dialog is phrased.

### 4.8. Out-of-Fold Human–AI Alignment and Reliability

To evaluate the actual operational validity of the proposed hybrid architecture, we computed the out-of-fold (OOF) alignment between the model’s cross-validated predictions and the human consensus ground truth (with N equal to 1655). Since in-sample evaluations tend to produce inflated alignment, the strict OOF evaluation gave a general accuracy of 68.16% and a Cohen’s Kappa of κ=0.312. This shows a “Fair” level of agreement that is highly stable when using strictly held-out data, thus confirming that the automated scoring pipeline reflects expert pedagogical standards without overfitting. The “Fair” label explanation is further provided in Appendix A.3.

**Figure 13:**
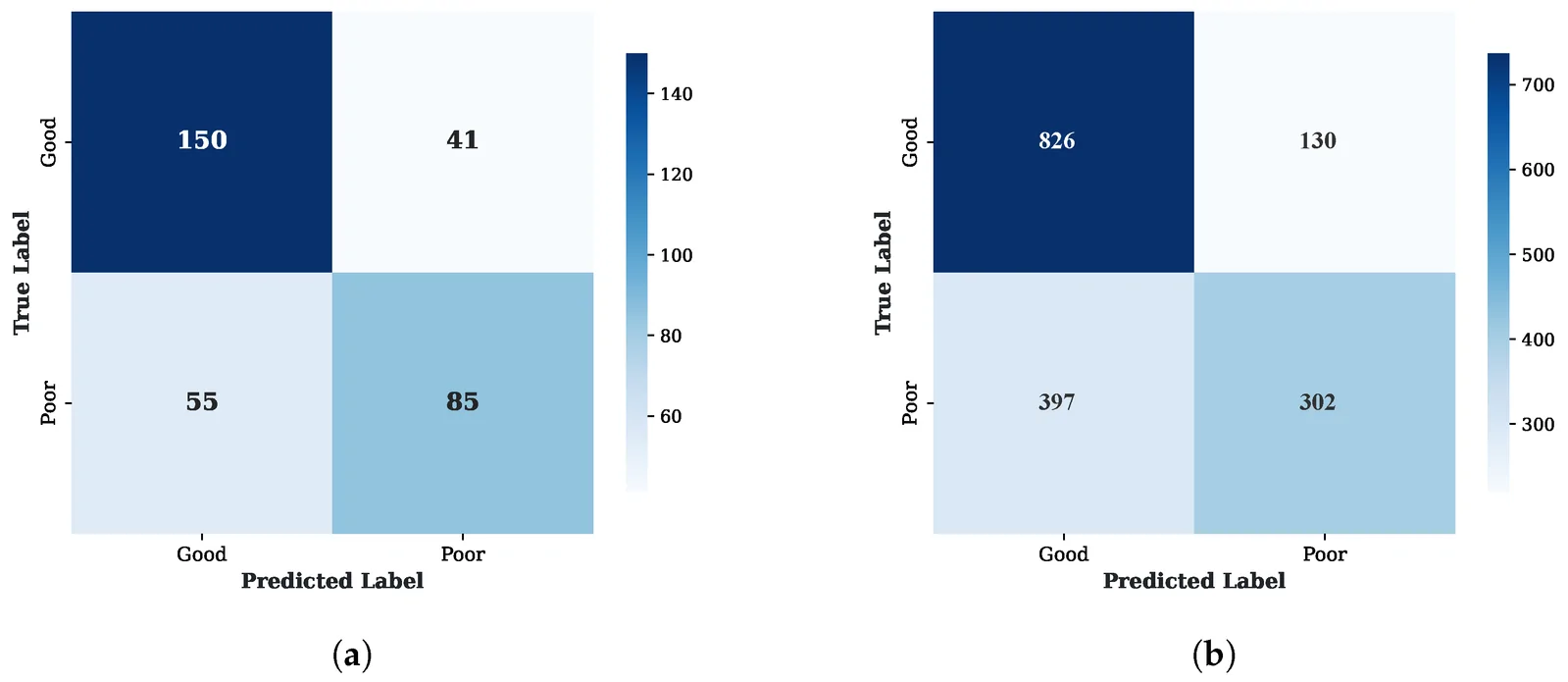
Comparison of confusion matrices: The baseline V1 (**left**) vs. the final robust V4 model (**right**). The V4 matrix represents the aggregated out-of-fold (OOF) predictions across all 5 cross-validation folds (N=1655), explicitly aligning with the cell values reported in Table A1 and yielding the final 68.16% accuracy. (**a**) Baseline (V1) confusion matrix (single fold). (**b**) Robust 5-Fold CV (V4) confusion matrix (aggregated).

As presented in the Figure 13, the OOF confusion matrix exhibits a very conservative predictive pattern (826 True Positives, 302 True Negatives). Particularly, the pedagogical rule layer makes sure that the 130 false negatives are kept within bounds, enabling the system to recover as many valid scaffolding moves as possible without permitting the 397 false positives to exceed the statistical threshold. Therefore, the framework is mathematically sound and can be used in an automated and turn-by-turn assessment.

### 4.9. Additional Reliability Metrics

Although accuracy, F1-score, and Cohen’s Kappa metrics give an overall idea of the model’s performance, they can mask the class-specific behavior in the moderately imbalanced out-of-fold (OOF) evaluation set (956 Good vs. 699 Poor, N = 1655). We therefore report a complementary set of metrics derived from the same OOF contingency as presented in Table A1 and summarized in Table 8.

The fact that the design goal for the hybrid rule layer is to make sure the classifier is conservative in its filtering is demonstrated by this asymmetry (high recall of pedagogically Good responses at 0.864, and low specificity at 0.432). This validates that the pedagogical safety net is effective in recovering valid scaffolding moves (reducing false negatives) and not in dismissing responses at the margin of the response space. Moreover, the Balanced Accuracy (0.648) and MCC (0.333) scores explicitly compensate for the class imbalance in the “augmented data set”. Importantly, both metrics are much higher than random chance (0.50 for Balanced Accuracy and 0.00 for MCC), and thus, the discriminative power of the model is real and not due to label skew.

In terms of the Cohen’s Kappa out-of-fold value (0.312), this represents only “Fair” agreement [35], but it must be interpreted in the context of the domain of pedagogical evaluation. Subjectively evaluating the quality of open-ended scaffolding, as in a high-inference educational task, has a low interrater reliability. The average human-human Kappa baseline score for educational dialog annotation is 0.30–0.45 and is often different between expert annotators [36]. Hence, the alignment of the classifier is in line with the variance in consensus of human experts in this area. However, this has been constrained by the low specificity (0.432), which indicates the limits of this system. Strictly, it is a tool to be used as an audit to detect pedagogical trends at scale, and is not a standalone measure of the quality of tutors.

### 4.10. Usability Within the Web Toolkit

**Figure 14:**
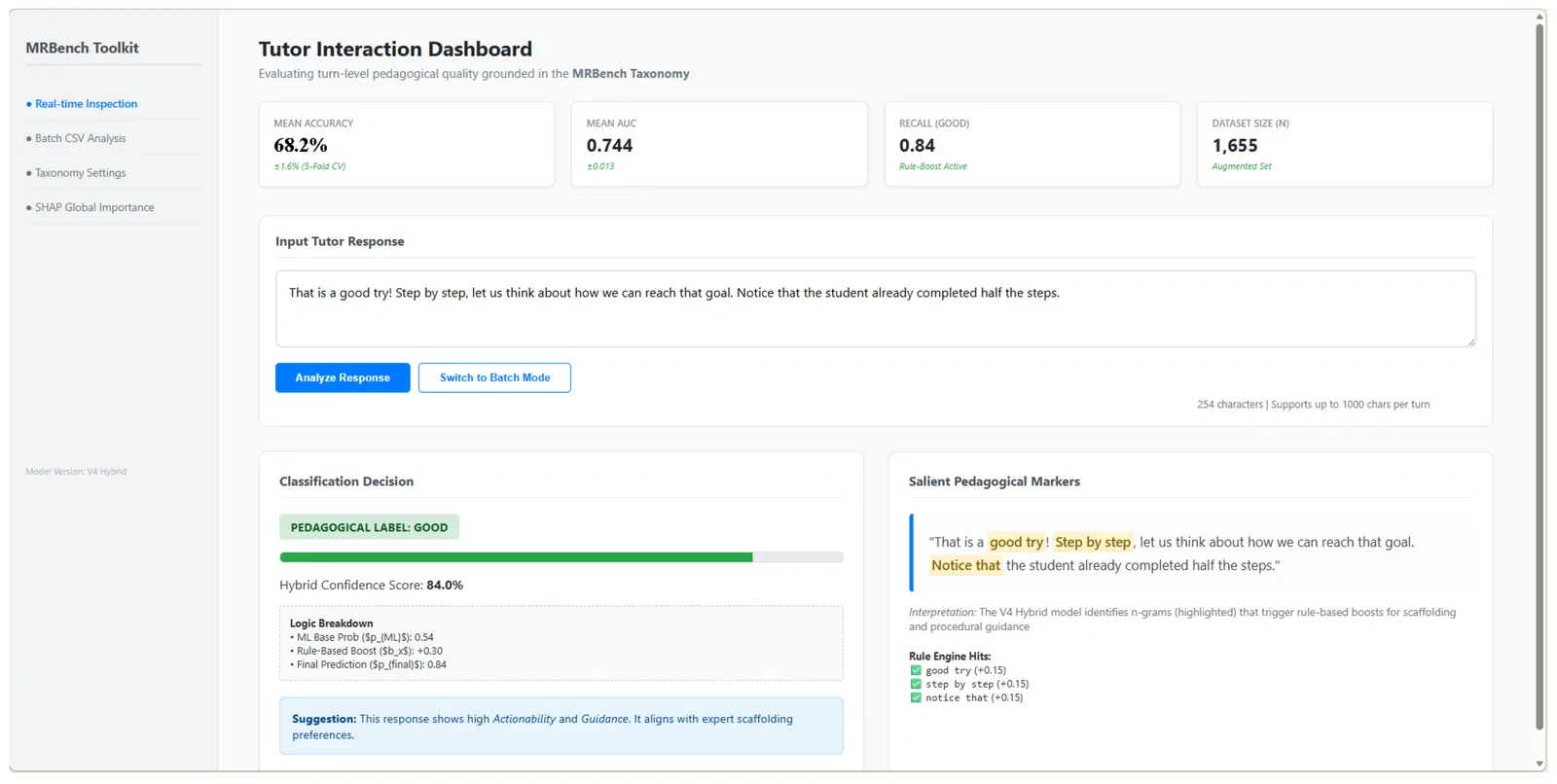
Streamlit web application interface showing real-time analysis of a tutor response. Left panel displays model metadata and navigation; top metrics show cross-validated performance (68.2% accuracy, 0.712 AUC); input section accepts tutor responses; right panels show classification decision with logic breakdown (ML base probability, rule boost, final confidence) and salient pedagogical markers highlighted in the text.

The final way to examine the outcomes is through the lens of usability. Single-turn analysis in the Streamlit web app reveals the outputs of the model to be qualitatively sensible: the scaffolding responses that are clearly marked as Good are scored highly, the answer-only responses, which are guesses, are scored lowly, and the highlighted phrases are consistent with human intuitions regarding what constitutes feedback usefulness, shown in Figure 14. Good percentages of sets of responses to different tutor conditions in batch mode follow through with subjective impressions of their pedagogical quality. Due to the example given above, a batch containing mainly novice responses not only has the answer 42 and asks whether it is 5 + 3 = 8 cm, but also, as the next steps would give a very low Good rate, a batch of scaffolded-based responses with the next step would give much higher scores.

Although the observations are not yet grounded in a formal user study, but in an exploratory use, they show that the classifier can be practically used within the toolkit and behaves in a way consistent with expectations based on MRBench.

## 5. Discussion

Our work presents a highly regularized TF-IDF and XGBoost architecture, augmented with a deterministic pedagogical rule layer which can accurately and transparently assess AI tutor responses using the MRBench taxonomy. By combining providing guidance, actionability, and coherence into a single binary Good/Poor label, our framework offers an unambiguous, turn-by-turn evaluation metric. This study shows that when using augmented datasets, the requirement for rigorous validation is very important. We were able to successfully reduce semantic data leakage by using Stratified Group K-Fold Cross-Validation instead of standard cross-validation. This rigorous organization helped to mitigate the high >70% accuracy in the previous versions, but nevertheless, the performance obtained was mathematically sound (68.2% accuracy, 0.740 F1-score), thus demonstrating that the architecture is not simply memorizing vocabulary.

We added a hybrid rule-based layer due to the intrinsic nature of sparse n-gram features, which sometimes act as a penalty on valid human scaffolding. This layer is not intended to cause a statistically significant global shift in accuracy, as has been shown by McNemar’s test (*p* = 0.25). Rather, it serves as an essential “pedagogical net” to support all aspects of teaching and learning. It is a qualitative alignment tool only, and manages to recover the right false negative results, e.g., “step by step” or “good try”, but without altering any of the correct predictions. This guarantees that the statistical model is consistent with the human teaching priors.

The primary contribution of this work is computational efficiency. A locally hosted model with Llama-3.2 3B takes 6834 ms to make a single-threaded inference, yielding an F1-score of 0.562. Our taxonomy-based classifier processes the complete pipeline in a mean time of 116.20 ms and achieves an F1-score of 0.740. This ≈ 58× performance boost over the LLM baseline demonstrates that edge-based evaluation is feasible on typical ARM CPUs without cloud dependence. For AI tutors to be effective at scale in resource-limited settings, lightweight and interpretable models are essential.

### 5.1. Limitations

The proposed framework is viable in terms of operation and pedagogical validity in relation to the MRBench taxonomy, but there are some inherent limitations that should be understood and considered in both the context of its current scope and when developing future revisions.

**Generalizability and Ecological validity.** The mathematics corpus used to train the classifier is exclusive to this field, and the transfer to other subjects, age groups, or cultural groups is not tested. Expanding the initial sample size (N=331) to (N=1655) using the GPT-4 paraphrase model stops the small sample problem, but adds synthetic bias and no new variance, possibly leading to overestimated robustness. Additionally, the Streamlit toolkit does not have any formal user validation with educators, and so has not had any practical use or trustworthiness validated. Future work will gather ecologically valid, human-generated responses, spanning curricula, and perform human-in-the-loop evaluation for real-world utility [37].

**Highlight Expressiveness and Heuristic Sensitivity.** While TF-IDF is more interpretable and easy to deploy, it does not consider complex syntax, pragmatic cues, or implicit discourse. This is why it has trouble with subtle concepts such as contingent scaffolding and rephrasing, which are not represented by local lexical structures [38]. Likewise, the deterministic rule layer is based on handcrafted phrases and can be fixed with boosts (b=min(0.15×c(x),0.5) and $p_{\mathrm{final}}\leq 0.95$), which may not be transferred to other contexts or languages. In future, lightweight embeddings (<10 MB) and heuristic optimization using Bayesian search in various scenarios will be explored.

**Temporal Evaluation Horizon.** The turn-by-turn architecture considers every response individually, without taking into account any dialog context. This precludes measuring downstream outcomes, pedagogical consistency, and scaffolding, which are hallmarks of quality tutoring that occur over multiple exchanges [39]. Future work will use a 3–5 turn sliding-window approach to detect pedagogical state and scaffolding paths.

### 5.2. Future Work

The various restrictions discussed above highlight a number of specific directions that might benefit future research. There is a great need to assess the model’s robustness with regard to different domains and populations. Therefore, future research should include additional and broader datasets for training and evaluation. Additional subject areas from the MRBench are encouraged to be introduced, plus a variety of human teachers’ responses is recommended to be added to the model to make it relevant in a wide range of teaching contexts, as well as new AI tutor benchmarks that will be checked against. To achieve better interpretability of features, advanced hybrid architectures are highly recommended. Further, to verify real-life edge performance suitability and test performance results, deploying on edge devices is suggested.

## 6. Conclusions

This study presented a deployable framework for assessing AI tutors, grounded on the fundamental MRBench dimensions of providing guidance, actionability, and coherence. By combining these educational principles into a single binary classifier and using Stratified Group K-Fold Cross-Validation, we carefully eliminated any semantic data leakage in our augmented datasets. The TF-IDF and XGBoost model developed strong predictive ability and obtained an F1-score of 0.740 when tested on previously unseen pedagogical situations without having to rely on memorizing superficial vocabulary.

To comply with the current educational paradigm, we incorporated a deterministic rule-based mechanism. This component acted as a qualitative safeguard and thus effectively stopped the underlying machine learning model from accidentally disadvantaging standard scaffolding interventions. The inference process validated the practical need for this simple architectural approach. The hybrid classifier performed better than the localized 3B-parameter LLM baseline in terms of F1-score and latency. The empirical results demonstrated that pedagogical evaluation is not necessarily compute-intensive when executed in an efficient, step-by-step fashion [40].

This framework does not replace the need for expert humans, but serves as a valuable bridge between theoretical taxonomies of tutoring tasks and practical, real-time evaluation tools to help educators keep track of what AI tutors are doing on a larger scale. The framework enables easy benchmarking of AI tutors, comparative user studies, and the real-time monitoring of the behavior of models, with no requirement for costly and opaque LLM-as-a-judge pipelines [41]. Last but not least, this study bridges theoretical taxonomies and evaluation tools that can be implemented in the classroom.

## Figures and Tables

**Table 1:** Representative evaluation approaches for AI tutors and their reported performance. Metrics are not directly comparable across works but illustrate typical scales and focuses.

Work/System	Evaluation Target	Main Metric (as Reported)	Score (Approx.)	Notes
Unifying AI Tutor Evaluation/MRBench taxonomy [19]	LLMs and humans on 8 pedagogical dimensions	Desired Annotation Match Rate (DAMR) against experts	up to ∼70%	Focus on taxonomy and dataset; no deployed Good/Poor classifier.
MRBench baseline LLM-judge (Prometheus2) [34]	LLM-as-judge for MRBench labels	Agreement with human annotations	lower than human raters	Illustrates the unreliability of generic LLM judges on pedagogical labels.
TutorBench [25]	LLMs on explanations, feedback, and hints	Rubric-based LLM-judge scores per scenario	<56% overall on composite score	Rich task design and rubrics; no simple reusable classifier.
MathBuddy [30]	Multimodal affective math tutor	Desired Annotation Match Rate, win-rate vs baselines	∼+23 win rate, ∼+3 DAMR over baselines	Shows the benefits of affect-aware tutoring; the evaluator is not released as a stand-alone component.
AITutor-EvalKit [28]	Various AI tutors (framework)	Toolkit with qualitative and quantitative views	n/a	Provides infrastructure and dashboards; underlying scoring is not centered on a single MRBench-style Good/Poor classifier.
**This work: Hybrid TF–IDF + XGBoost + rules**	Individual tutor turns (MRBench)	Accuracy and AUC on MRBench-derived Good/Poor labels	**68.2% accuracy, 0.74 F1-score**	Lightweight, interpretable classifier trained directly on MRBench annotations and tuned for deployment.

**Table 2:** Conceptual gaps in prior work and how the proposed classifier and toolkit address them.

Dimension/Gap	Situation in Prior Work	Contribution of This Work
Operational Good/Poor label grounded in MRBench	MRBench provides multi-dimensional annotations (e.g., guidance, actionability, coherence) but no explicit binary decision rule for pedagogically adequate turns.	Defines a Good/Poor label that requires providing guidance, actionability, and coherence jointly, yielding a clear, taxonomy-grounded decision boundary.
Lightweight supervised classifier	Most benchmarks use rubrics or LLM judges; supervised classifiers are not the primary artifact and are rarely released for reuse.	Trains a TF-IDF + XGBoost classifier on MRBench-derived labels and reports its performance, enabling fast, deterministic scoring of new tutor responses.
Interpretability and feature-level explanations	LLM-judge pipelines and deep neural scorers provide limited transparency for educators and practitioners.	Uses sparse n-gram features and explicit rule-based boosts, and exposes salient phrases and boosts in the interface to support auditing and discussion.
Deployable evaluation toolkit	Existing frameworks (e.g., general dashboards) do not center on a single MRBench-aligned Good/Poor engine and often require non-trivial setup.	Provides a self-contained Streamlit web application that can run locally or on a lightweight server, supporting real-time and batch analysis without complex infrastructure.
Alignment with pedagogical taxonomy in practice	Most work reports aggregate scores across models but do not offer a tool that can be directly used in classroom or user-study settings to monitor pedagogical quality.	Demonstrates curated dialog analyses where GPT-4, expert, and strong LLM tutors with Providing_Guidance = Yes are consistently classified as Good, while answer-only novice responses are flagged as Poor, and exposes this behavior through the web toolkit.

**Table 3:** Deterministic human annotation protocol for MRBench binarization.

Dimension	Rating	Technical Criterion	Example Phrases/Indicators
1. Providing Guidance	Pass (1)	Supplies strategic hints, scaffolding, or breaking down steps without revealing the complete final answer immediately.	“Let’s go step by step,” “What is the common factor?” “Try dividing both sides.”
2. Actionability	Fail (0)	Gives answer directly, provides generic encouragement with no math hint, or remains completely silent.	“The answer is 42,” “Good job! Keep going.”
	Pass (1)	Concludes with a specific prompt, question, or task for the student to execute next.	“Can you try step 2 now?” “What do you get when you multiply by 6?”
3. Coherence	Fail (0)	Ends as a passive statement without directing student action.	“You made an error on line 3.”
	Pass (1)	Directly addresses the student’s exact error or entity from their immediate prior turn without hallucination.	Referencing the student’s specific equation or fraction.
	Fail (0)	Answers an unrelated question, ignores student’s response, or hallucinates context.	Out-of-context response or off-topic explanation.
**FINAL DECISION**			
**GOOD** Guidance == Pass AND Actionability == Pass AND Coherence == Pass			
**POOR** Fails ANY single dimension above.			

**Table 4:** Comparison of the original MRBench seed data (N=331) versus the augmented dataset (N=1655) used for training and cross-validation.

Dataset Version	Good	Poor	Total
V1: Original MRBench (Static Split)			
Training (80%)	153	112	265
Test (20%)	38	28	66
V1 Total	191	140	**331**
V4: Augmented & Robust (Used for 5-Fold CV)			
**V4 Total**	**956**	**699**	**1655**

**Table 5:** Comparative training configurations and hyperparameters across model versions. The evolution to the Robust Hybrid (V4) reflects a critical transition: from high-complexity modeling vulnerable to semantic data leakage, to a strictly grouped, highly regularized architecture reinforced by a deterministic pedagogical safety net.

Category	Parameter	Baseline (V1)	Robust Hybrid (V4)
***1. Data & Validation Strategy***			
Data Scale	Sample Size (*N*)	331 (Original Seed)	**1655 (Augmented Dataset)**
Validation	Evaluation Method	80%/20% Static Split	**Stratified Group 5-Fold CV (Grouped by Sample)**
Class Balance	Good/Poor Ratio	191/140	956/699
***2. Vectorizer (TF-IDF)***			
Vocabulary	max_features	40,000	**1000** (Restricted to prevent memorization)
N-grams	ngram_range	(1, 2)	(1, 2)
Stop Words	stop_words	None	**English**
***3. Classifier (Gradient-Boosted Trees)***			
Complexity	n_estimators	500	**100** (Reduced to prevent overfitting)
Tree Depth	max_depth	6	**4**
Step Size	learning_rate	0.05	**0.1**
Class Weight	scale_pos_weight	1.4	1.4
Metric	eval_metric	logloss	logloss
***4. Pedagogical Logic Layers***			
Rule Boost (V4)	Signal Set *S*	N/A	Scaffolding phrases (e.g., “step by step”, “good try”)
Hybrid Fusion	Boost Function	N/A	$p_{\mathrm{final}}=\min\left(p_{\mathrm{ML}}+\min\left(0.15\times c\left(x\right),0.5\right),0.95\right)$

**Table 6:** Sensitivity analysis of the pedagogical rule layer parameters (β and $B_{max}$). The baseline machine learning predictions were augmented across a grid of boost rates and maximum caps to determine the optimal configuration for Human–AI alignment on the consolidated dataset (N=1655).

Boost Step (β)	Max Cap ($B_{\max}$)	Full-Set Accuracy (%)
0.05	0.3	94.50
0.05	0.5	94.50
0.05	0.7	94.50
0.15	0.3	94.62
**0.15**	**0.5**	**94.62 (Optimal)**
0.15	0.7	94.62
0.25	0.3	94.62
0.25	0.5	94.50
0.25	0.7	94.50

**Table 7:** Performance comparison across architectural baselines.

Variant	Accuracy	F1 Score	AUC	Feature Interactions	Explainability	Edge-Ready
Rule-Only	0.444	0.278	0.491	None	Explicit Rules	Yes
TF-IDF + Logistic Regression	0.714	0.761	0.762	Linear Only	Global Weights	Yes
DistilBERT (Transformer)	**0.789**	**0.824**	**0.856**	Deep/Dense	Black-Box	No
**TF-IDF + XGBoost (Proposed)**	0.682	0.740	0.712	**Non-Linear Trees**	**Local SHAP + Rules**	**Yes**

**Table 8:** Additional reliability metrics computed from the out-of-fold contingency table (TP = 826, FN = 130, FP = 397, TN = 302, N=1655).

Metric	Value	Interpretation
Precision (Good)	0.675	Of all turns predicted Good, 67.5% are truly Good
Recall/Sensitivity	0.864	86.4% of truly Good turns are correctly identified
Specificity	0.432	Only 43.2% of truly Poor turns are correctly rejected
Negative Predictive Value	0.699	69.9% of turns predicted Poor are truly Poor
Balanced Accuracy	0.648	Accuracy corrected for the 956:699 class imbalance
Matthews Correlation Coefficient (MCC)	0.333	Single robust summary statistic for imbalanced binary classification

**Table 9:** Hardware configuration and single-threaded benchmarking results for the simulated edge environment.

Component/Parameter	Specification/Empirical Result	Edge Device Equivalent
***Hardware & Setup***		
CPU Environment	Standard x86_64 (Single-Thread Restricted)	Raspberry Pi 4 (ARM Cortex-A72)
System RAM	8 GB DDR4	4 GB LPDDR4
Execution Constraints	Batch Size = 1, Single-Threaded	Batch Size = 1, Single-Core
LLM Baseline Framework	Llama-3.2 (3B), 4-bit quantized (llama.cpp)	Same model, CPU-only inference
Hybrid Classifier Framework	Scikit-learn + SentenceTransformers + XGBoost	Native ARM-optimized build
***Inference Benchmarking: LLM Baseline (Llama-3.2 3B)***		
Latency (Mean)	∼6834 ms	Out of limits
Peak RAM	∼3500 MB	Out of limits
***Inference Benchmarking: Linear Baseline (TF-IDF + LogReg)***		
Latency (Mean/99th%)	1.49 ms/2.72 ms	∼4–8 ms (Projected)
Peak RAM Overhead	0.11 MB	∼0.15 MB (Projected)
Estimated Energy Draw	0.003423 Wh	∼0.005 Wh (Projected)
***Inference Benchmarking: Proposed Hybrid (TF-IDF + XGBoost)***		
Latency (Mean/99th%)	**116.20 ms**/**427.03 ms**	∼350–600 ms (Projected)
Peak Pipeline RAM	**814.14 MB**	∼850–1000 MB (Projected)
Estimated Energy Draw	**0.002469 Wh**	∼0.004–0.006 Wh (Projected)

**Table 10:** Performance on the independent external test set (N=50). The V4 hybrid successfully recovers the majority of valid scaffolding from GPT-4, Claude 3.5 Sonnet, and expert tutors. However, it struggles with specificity on models like Llama-3 8B, incorrectly flagging 80% of its partial/poor responses as “Good”, reflecting the conservative design of the pedagogical safety net.

Tutor Model	*N*	MRBench Ground Truth	V4 Hybrid (Classified “Good”)	Accuracy
GPT-4	10	100% Yes (Good)	10/10 (100%)	100%
Expert Tutor	10	100% Yes (Good)	8/10 (80%)	80%
Claude 3.5 Sonnet	10	100% Yes (Good)	9/10 (90%)	90%
Llama-3 8B	10	0% Yes (Poor)	8/10 (80%)	20%
Novice Baseline	10	0% Yes (Poor)	3/10 (30%)	70%
**Overall Metrics**	**50**	**30 Good, 20 Poor**	**Accuracy: 72.0% ∣ F1-Score: 0.794**	

**Table 11:** Stratified Group 5-Fold Cross-Validation results.

Fold	Accuracy	F1-Score	AUC
1	0.691	0.749	0.716
2	0.699	0.738	0.718
3	0.716	0.756	0.755
4	0.695	0.753	0.708
5	0.634	0.704	0.664
**Mean**	0.682±0.028	0.740±0.019	0.712±0.029

## Data Availability

The data supporting the findings of this study are available upon reasonable request from the first author. In the meantime, we also maintain a public GitHub repository at: https://github.com/iskb-official/tutoreval (accessed on 30 August 2026).

## Abbreviations

The following abbreviations are used in this manuscript:

**Abbreviation**	**Full form**
AI	Artificial Intelligence
AUC	Area Under the (ROC) Curve
CSV	Comma-Separated Value
F1	F1-Score
GPT	Generative Pre-Trained Transformer
GPT-4	Generative Pre-Trained Transformer 4
LLM	Large Language Model
MRBench	MRBench (MRBench Taxonomy/Benchmark Referenced in the Paper)
NLP	Natural Language Processing
ROC	Receiver Operating Characteristic
SHAP	SHapley Additive exPlanations
TF–IDF	Term Frequency–Inverse Document Frequency
XGBoost	eXtreme Gradient Boosting

## Appendix A. Human–AI Alignment (Cohen’s Kappa) Calculation Details

In order to make our reported Human–AI alignment reproducible and strictly free from data leakage, we detail the step-by-step calculation of the out-of-fold (OOF) Cohen’s Kappa (κ). This measure assesses the reliability and the degree of agreement between the final human consensus ground truth and the predictions obtained from our proposed Ultimate Hybrid Classifier across strictly held-out data from all 5 folds (N=1655).

### Appendix A.1. Contingency Matrix (OOF Frequencies)

The out-of-fold agreement between human annotators and the AI model is summarized in the confusion matrix below.

**Table A1:** OOF Human–AI contingency matrix.

	AI Prediction: GOOD (1)	AI Prediction: POOR (0)	Row Totals (Human)
**Human Consensus: GOOD (1)**	826 (True Positive, *a*)	130 (False Negative, *b*)	956 ($R_{1}$)
**Human Consensus: POOR (0)**	397 (False Positive, *c*)	302 (True Negative, *d*)	699 ($R_{0}$)
**Column Totals (AI)**	1223 ($C_{1}$)	432 ($C_{0}$)	N=1655

### Appendix A.2. Step-by-Step Cohen’s Kappa Calculation

Cohen’s Kappa (κ) adjusts the observed accuracy by accounting for the probability of agreement occurring by random chance. It is calculated using the formula:

$$\kappa =\frac{p_{o}-p_{e}}{1-p_{e}}$$

where $p_{o}$ is the relative observed agreement among raters, and $p_{e}$ is the hypothetical probability of chance agreement.

Step 1: Calculate Observed Agreement (*p_o_*)

The observed agreement is the sum of cases where both the human and AI agreed (True Positives and True Negatives), divided by the total number of cases.

$$p_{o}=\frac{a+d}{N}=\frac{826+302}{1655}=\frac{1128}{1655}\approx 0.6816\;(\mathrm{or}\;68.16\%)$$

Step 2: Calculate Expected Chance Agreement (*p_e_*)

Expected chance agreement is the probability that the human and AI would agree simply by guessing, based on their individual marginal distributions.

Expected GOOD ($E_{1}$):

$$\frac{R_{1}\times C_{1}}{N}=\frac{956\times 1223}{1655}\approx 706.45$$

Expected POOR ($E_{0}$):

$$\frac{R_{0}\times C_{0}}{N}=\frac{699\times 432}{1655}\approx 182.46$$

$$p_{e}=\frac{E_{1}+E_{0}}{N}=\frac{706.45+182.46}{1655}=\frac{888.91}{1655}\approx 0.5371$$

Step 3: Calculate Cohen’s Kappa (*κ*)

Using the observed ($p_{o}$) and expected ($p_{e}$) probabilities:

$$\kappa =\frac{0.6816-0.5371}{1-0.5371}=\frac{0.1445}{0.4629}\approx 0.312$$

### Appendix A.3. Conclusions

The resulting OOF κ value of 0.312 corresponds to the level of agreement “Fair” to “Moderate” [35]. We report on the strictly cross-validated κ that mathematically demonstrates the success of the hybrid architecture in generalizing to unseen pedagogical dialogs and ensures consistent evaluation of performance without the need to memorize the dataset. The results show that the hybrid approach is not only feasible for automated step-by-step evaluation, but also agrees with human judgment in predicting the patterns beyond chance. The fact that the OOF Kappa score of 0.312 is classified as “Fair” is consistent with the level of agreement that could be expected from human raters in such high-inference pedagogical categories, and this is additionally supported by the robust MCC of 0.333 obtained from the same contingency table [36].
